# Spatial connectivity pattern of expanding gilthead seabream populations and its interactions with aquaculture sites: a combined population genetic and physical modelling approach

**DOI:** 10.1038/s41598-019-51256-z

**Published:** 2019-10-11

**Authors:** Iva Žužul, Tanja Šegvić-Bubić, Igor Talijančić, Tomislav Džoić, Ivana Lepen Pleić, Gordana Beg Paklar, Stjepan Ivatek-Šahdan, Ivan Katavić, Leon Grubišić

**Affiliations:** 1 Institute of Oceanography and Fisheries, PO Box 500, Šetalište Ivana Meštrovića 63, 21000 Split, Croatia; 2 Meteorological and Hydrological Service of Croatia, Grič 3, Zagreb, Croatia

**Keywords:** Population genetics, Biological physics

## Abstract

In gilthead seabream the number of domesticated individuals increased annually, and escape events occur regularly in the Adriatic Sea. Still there is a lack of population genetic characteristics and evidence of the extent and geographic scale of interbreeding resulting from fish-farm escapees. We screened 1586 individuals using a panel of 21 neutral microsatellite loci in several consecutive years and here report on the medium-scale detection of hybrid and farmed seabream in the natural environment. Wild adults showed a lack of genetic structure within basin and sampling years and reduced connectivity with wild offspring collection, suggesting their temporal residency within the Adriatic. On the contrary, by linking the results of multiannual genetic analyses with the results of coupled hydrodynamic and individual based models (IBM-Ichthyop), we observed a strong connection of wild seabream associated with tuna-aquaculture sites and offspring from the nursery grounds, indicating that the surroundings of tuna sea-cage farms can function as a spawning grounds. The study results present the genetic baseline of wild and farmed strains from the eastern Adriatic Sea, as a first step toward development of a mitigation strategy for fish escapees aimed at controlling further erosion of genetic integrity.

## Introduction

The gilthead sea bream *Sparus aurata*, like the European seabass *Dicentrarchus labrax*, has been the object of intensive farming and has become the most important marine farmed fish in the Mediterranean^1^. Following EU trends, gilthead sea bream production in Croatia has increased at a rate of 65% in the past ten years. However, production is still relatively small compared to the rest of the Mediterranean region (4,304 *vs*. 160,563 tonnes in 2016)^2^.

Interestingly, in recent years, a significant increase of wild gilthead seabream has been documented in coastal areas of the Adriatic Sea^3,4^, and elsewhere in the Mediterranean, such as Greece^5^. Along the eastern Adriatic coast, the highest gilthead seabream landings are noted in late autumn (November-December) in the pre-spawning aggregation period of species, reaching daily catches of several tonnes per purse-seins vessel (pers. comm.). Highly abundant and seasonally stable aggregations of gilthead seabream were observed around semi-offshore tuna farms, and displayed enhanced fitness status due to the high energy trophic resources found at farming sites^6,7^. In addition, shellfish farms become also very attractive food source sites for seabream populations^3^. High mussel losses due to gilthead seabream predation have been reported at shellfish farms along the Adriatic coast, significantly affecting the economic stability of this industry^8^.

It has been suggested that the rapid expansion of sea-cage farming facilities contributed to the population increase through the escape of fish from sea-cage aquaculture and/or escape of viable, fertilized eggs spawned by farmed fish inside sea-cages, i.e. escapes through spawning^4,9–13^. Escaped fish have been shown to have detrimental effects on native fish stocks, due to competition for resources, spread of disease, and alteration of genetic diversity due to hybridisation^14^. Hybridization events may break down locally adapted gene complexes, reduce competitive ability and overall fitness of wild populations^15^. The impact may be more enhanced in cases when farmed individuals are of a different geographical origin, as is the case in Croatian aquaculture, and/or have experienced strong domestication^9,16^.

On the other hand, ocean warming may also be behind the increasing gilthead seabream abundance. This is currently one of the main driving forces causing changes in species abundance and distribution and, thus, in species biodiversity and functioning of marine ecosystems^17–20^. Annual trend distribution of the Mediterranean sea surface temperature (SST) indicates a warming trend throughout the basin with average values of 0.035 ± 0.007 °C per year^21^. Thus, it could be argued that wild gilthead seabream, as a subtropical Sparid, is taking advantage of the present climate change in terms of increased larval survival and subsequent recruitment success, but also in establishment at the northern limits of distribution areas (e.g., Brittany and Denmark coast)^3,22,23^.

To date, molecular markers have been successfully employed in the discrimination of fish origins and in identifying hybridization processes among wild and farmed fish^4,9,24–28^. In the case of gilthead seabream, the application of molecular markers is a challenge in detecting escapees and hybrids, since (i) gilthead seabream have been domesticated relative recently, i.e. have a weak selection footprint^29^; (ii) the high dispersal capacity of this species produces a high exchange of migrants among subpopulations, allowing a large effective subpopulation size and low structuring, and (iii) historical samples are not available for comparison analyses.

To explore the genetic population structure and footprints of selection in gilthead seabream populations of different origin (wild vs. farmed) and ontogenetic state (juveniles vs. adults) from the Adriatic Sea, we genetically assayed 1586 individuals with 21 putatively neutral microsatellites^30^. Thus, this study aimed to: (i) examine the possible changes in genetic variation occurring over the spatial and short-term temporal scale of wild seabream populations from the eastern Adriatic, with special attention on populations sampled around fish farms and their potential impact on recruitments from surrounding nursery grounds, (ii) investigate the genetic structure of farmed populations originating from different hatchery sources and (iii) quantify the presence and magnitude of hybridization between wild and escaped farmed individuals.

## Results

### Genetic diversity

**Figure 1:**
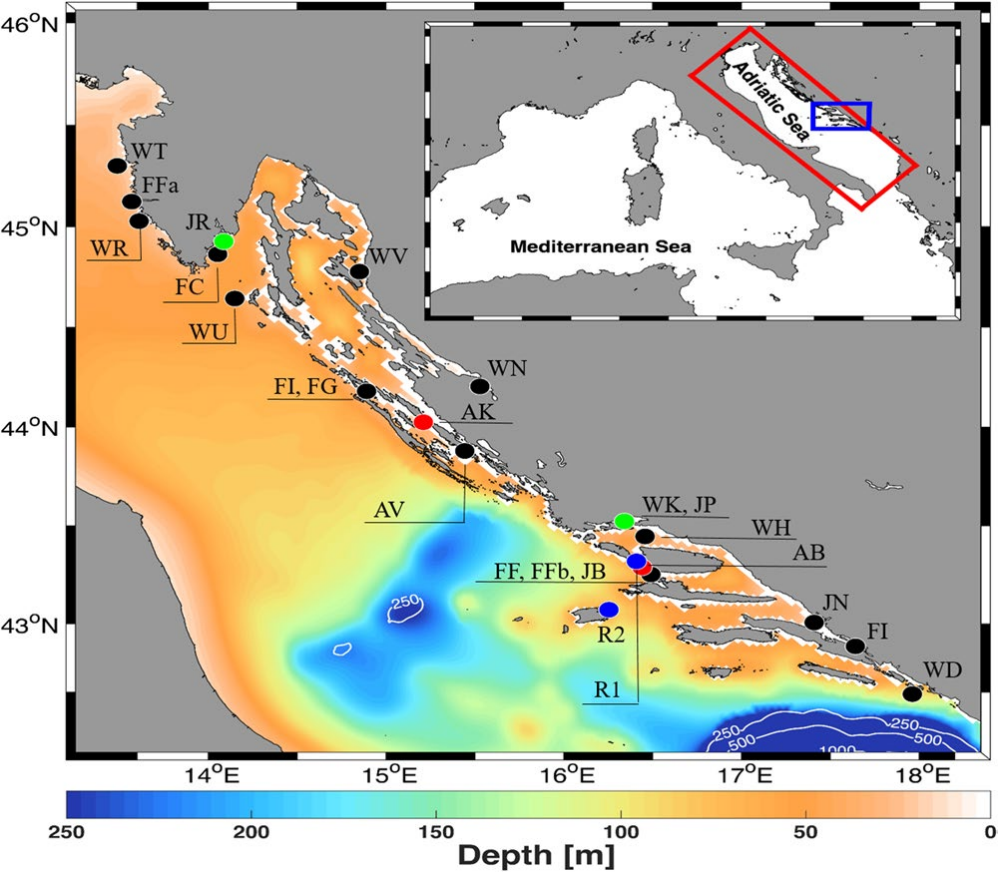
Adriatic Sea bathymetry with sampling locations of wild adults gilthead seabream (WH, Brač channel; WT, Tar Bay; WN, Novigrad Sea; WR, Rovinj; WU, Unije; WV, Velebit Channel; WD, Dubrovnik), wild young-of-the year (YOY) (JP, Pantana River; JN, Neretva River; JR, Raša River; JB, Maslinova Bay), farm-associated adults (AK, Mrdina; AB, Maslinova Bay; AV, Vrgada) and farmed adults (FC, Budava Bay; FF-FFb, Maslinova Bay; FI, Mali Ston Bay; FI-FG, Lamljana Bay; FFa, Lim Bay). Tuna-aquaculture sites (red dots) and nurseries (green dots) were involved in spatial particle distribution simulation. Blue points represent locations of HF radars at Cape Ražanj, island of Brač (R1 43.319688°N, 16.408743°E), and at Cape Stončica, island of Vis (R2; 43.072141°N, 16.254157°E). Contours are drawn for 250, 500 and 1000 m depths. On the map of the Mediterranean Sea (upper right corner), the Adriatic ROMS domain is shown in the red rectangle, and the ASHELF2 ROMS domain in the blue rectangle. More information about population abbreviations and sampling years are provided in Table 1. The figure has been created using MATLAB 2014a (www.mathworks.com) and GIMP 2.8.16 (www.gimp.org) software.

A total of 1586 individuals of *Sparus aurata* were genotyped at 21 neutral microsatellite loci (Fig. 1; Tables 1 and S1) where the proportion of missing data per locus ranged from 0 to 1.5%, with an average of 0.3%, respectively.

**Table 1:** Information of sampling locations, year and code, along with the origin and number of individual gilthead seabream that were genetically assayed with 21 putatively neutral microsatellites.

Location	Genotyped ind	Sampling year	Pop ID	Fish origin	Latitude	Longitude
***Wild adults***						
Brač channel	41	2009	09WH	W	43.40617	16.40367
Tar Bay	80	July, 2015	15WT	W	45.31303	13.6028
Novigrad Sea	90	July, 2015	15WN	W	44.20304	15.52879
Rovinj	89	February, 2016	16WR	W	45.04067	13.64931
Unije	47	February, 2016	16WU	W	44.63822	14.22177
Velebit Channel	86	February, 2016	16WV	W	44.77667	14.85083
Kastela Bay	29	February, 2016	16WK	W	43.52282	16.34182
Dubrovnik	19	February, 2016	16WD	W	42.63626	17.99329
***Wild YOY***						
Pantana River	124	May, 2015	15JP	W	43.52282	16.34182
Neretva River	71	May, 2015	15JN	W	43.05228	17.43431
Pantana River	25	May, 2016	16JP	W	43.52282	16.34182
Raša River	34	May, 2016	16JR	W	45.03181	14.04697
Maslinova Bay*	30	June, 2016	16JB*	W	43.29634	16.46636
***Farm-associated***						
Mrdina	56	February, 2015	15AK	WA	44.17946	14.89085
Maslinova Bay	44	February, 2015	15AB	WA	43.30077	16.44479
Vrgada	21	February, 2015	15AV	WA	43.87891	15.44309
Mrdina Bay	50	February, 2016	16AK	WA	44.17946	14.89085
Maslinova Bay	46	February, 2016	16AB	WA	43.30077	16.44479
Mrdina Bay	60	January, 2017	17AK	WA	44.17946	14.89085
Maslinova Bay	70	January, 2017	17AB	WA	43.30077	16.44479
***Farmed***						
Budava Bay	96	February, 2015	15FC	F-CRO	44.89079	13.99811
Maslinova Bay	97	March, 2015	15FF	F-FRA	43.29634	16.46636
Mali Ston Bay	57	March, 2015	15FI	F-ITA	42.8854	17.64069
Lamljana Bay	15	March, 2016	16FI	F-ITA	45.13217	15.21015
Lamljana Bay	71	March, 2016	16FG	F-GRE	45.13217	15.21015
Lim Bay	59	April, 2016	16FFa	F-FRA	45.13217	13.67286
Maslinova Bay	79	February, 2016	16FFb	F-FRA	43.29634	16.46636
Overall	1586					

W, wild; WA, wild adults associated with aquaculture sites; F, farmed originated from different fingerling suppliers (CRO, Croatia; FRA, France; ITA, Italy; GRE, Greece). The group marked with an asterisk (*) was sampled in the nursery ground located in the vicinity of an aquaculture area - Maslinova Bay.

Several populations showed significant deviation from Hardy-Weinberg equilibrium, with tendencies towards heterozygote deficiency at the loci M3, H8, G3 and L7 (Supplementary Table S2), as revealed by Fisher’s exact test. MICROCHECKER detected null alleles for these loci at estimated frequencies <0.3 for M3 and H8. Those two loci were removed from further analyses. Lower null allele frequencies <8% were detected at G3 and L7 and these loci were retained, since the estimation of *F*_ST_ both using and without using the ENA correction method gave comparable results; 0.022 *vs* 0.023 for *F*_ST_ with *vs* without the ENA, with overlapping 95% CI. There was no consistent evidence of linkage disequilibrium between any pair of loci following strict Bonferroni correction for multiple tests.

Among the 19 loci examined, all were polymorphic, with the number of alleles per locus ranging from 7 to 14. Significant positive correlations in allele frequencies between adjacent temporal wild populations sampled in 2009, 2015, and 2016 were recorded, suggesting stability in allele frequencies (Supplementary Table S3). Genetic diversity revealed varying degrees of genetic diversity among populations, ranging from 0.74 to 0.81 in expected heterozygosity (*H*e) (Table 2). Farmed gilthead seabream exhibited a significantly lower effective number of alleles per locus (6.5 *vs* 7.3), allelic richness (7.3 *vs* 8.6), observed (0.74 *vs* 0.76) and expected heterozygosity (0.79 *vs* 0.81) (ANOVA test, p < 0.05) in comparison to the wild counterparts. Wild young-of-the-year and farm associated populations followed the genetic diversity pattern observed for the wild group (Table 2). The inbreeding coefficient, *F*_IS_, ranged from −0.06 to 0.10 and was significantly higher than zero in 33% populations of the dataset (Table 2). Analyses of multilocus genotype data indicated strong disparity of contemporaneous effective population sizes (Ne) in respect to fish origin and ontogenetic state. On average, estimates of Ne were 3-fold, 7-fold and 17-fold smaller in the farmed group (164) in comparison to the wild group (549), farm-associated (1139) and wild YOY (2799), respectively.

**Table 2:** Summary statistics for genetic variation of gilthead seabream *Sparus aurata* in the Adriatic Sea showing the average number of alleles (A), effective number of alleles (Ae), allelic richness (Ar), expected (He) and observed (Ho) heterozygosity, fixation index (*F*_IS_) and effective population size (*N*_E_) for 21 putatively neutral microsatellite loci.

Pop ID	A	Ae	Ar	Ho	He	*F*_IS_	*N*_E_
**Wild**							
09WH	11.7 ± 6.1	6.4 ± 3.6	8.7 ± 3.8	0.77 ± 0.2	0.81 ± 0.1	0.05	565 (290, 6523)
15WT	13.3 ± 7.0	6.6 ± 3.9	8.8 ± 3.6	0.78 ± 0.1	0.80 ± 0.1	0.03	755 (475, 2002)
15WN	13.5 ± 6.9	6.7 ± 4.3	8.8 ± 3.8	0.77 ± 0.1	0.80 ± 0.1	0.04	∞ (3583, ∞)
16WR	13.7 ± 6.5	6.6 ± 3.8	8.7 ± 3.6	0.76 ± 0.1^*^	0.80 ± 0.1	0.05	5009 (953, ∞)
16WU	12.2 ± 5.9	6.1 ± 3.4	8.8 ± 3.5	0.76 ± 0.1	0.79 ± 0.1	0.05	29510 (587, ∞)
16WV	13.1 ± 6.7	6.8 ± 3.9	8.8 ± 3.6	0.77 ± 0.2^*^	0.81 ± 0.1	0.05^*^	6980 (1118, ∞)
16WK	9.7 ± 4.5	6.0 ± 3.3	8.4 ± 3.6	0.74 ± 0.2^*^	0.80 ± 0.1	0.07	—
16WD	9.7 ± 4.5	6.0 ± 3.1	8.9 ± 4.1	0.76 ± 0.2	0.81 ± 0.1	0.06	—
*Overall*	20.8 ± 12.7	7.3 ± 4.4	8.6^a^	0.76 ± 0.1^a^	0.81 ± 0.1^a^		549 (494, 616)
**Wild YOY**							
15JP	14.5 ± 7.9	6.8 ± 4.3	8.8 ± 3.7	0.75 ± 0.1^*^	0.81 ± 0.1	0.07^*^	1554 (626. ∞)
15JN	12.9 ± 6.3	6.4 ± 3.2	8.7 ± 3.4	0.76 ± 0.1^*^	0.81 ± 0.1	0.06^*^	900 (484, 5276)
16JP	9.9 ± 4.7	5.9 ± 2.8	8.5 ± 3.6	0.77 ± 0.2	0.80 ± 0.1	0.04	—
16JR	11.4 ± 4.8	6.4 ± 3.2	8.9 ± 3.6	0.76 ± 0.1	0.81 ± 0.1	0.07	1513 (365, ∞)
16JB	7.56 ± 3.4	4.4 ± 1.6	6.5 ± 2.6	0.72 ± 0.1	0.75 ± 0.1	0.04	108 (70, 218)
*Overall*	15.6 ± 8.2	6.8 ± 3.9	8.3^a^	0.75 ± 0.1^ab^	0.80 ± 0.1^a^		2799 (1452, 26309)
**Farm associated**							
15AK	12.4 ± 6.3	6.3 ± 3.4	8.6 ± 3.6	0.76 ± 0.2	0.81 ± 0.1	0.05	1430 (497, ∞)
15AB	11.8 ± 5.9	6.2 ± 3.5	8.7 ± 3.5	0.76 ± 0.1	0.81 ± 0.1	0.05	3435(484, ∞)
15AV	9.4 ± 4.0	5.4 ± 2.6	8.4 ± 3.3	0.74 ± 0.2	0.79 ± 0.1	0.06^*^	—
16AK	12.4 ± 5.9	6.6 ± 3.9	8.9 ± 3.7	0.75 ± 0.2^*^	0.81 ± 0.1	0.08^*^	925 (431, ∞)
16AB	11.8 ± 5.9	6.5 ± 3.8	8.7 ± 3.6	0.75 ± 0.1^*^	0.81 ± 0.1	0.07^*^	705 (384, 3637)
17AK	12.7 ± 6.6	6.7 ± 3.9	8.7 ± 3.8	0.74 ± 0.2	0.81 ± 0.1	0.08^*^	127 (105, 160)
17AB	12.9 ± 5.9	6.6 ± 4.0	8.7 ± 3.6	0.77 ± 0.1	0.81 ± 0.1	0.05^*^	521 (344, 1047)
*Overall*	16.1 ± 8.3	7.2 ± 4.6	8.7^a^	0.75 ± 0.1^ab^	0.81 ± 0.1^a^		1139 (899, 1534)
**Farmed**							
15FC	12.8 ± 6.8	6.5 ± 3.6	8.6 ± 3.6	0.77 ± 0.2^*^	0.80 ± 0.1	0.03	89 (82, 96)
15FI	11.3 ± 5.3	5.3 ± 2.6	7.5 ± 2.7	0.75 ± 0.2^*^	0.78 ± 0.1	0.04	76 (70, 83)
16FI	10.5 ± 4.7	4.9 ± 2.7	7.4 ± 2.8	0.73 ± 0.2^*^	0.76 ± 0.1	0.04	98 (83, 118)
16FG	7.4 ± 2.9	4.6 ± 1.8	7.4 ± 2.9	0.82 ± 0.2	0.78 ± 0.1	—0.06	—
15FF	8.9 ± 4.3	5.2 ± 2.6	6.8 ± 2.7	0.71 ± 0.1^*^	0.78 ± 0.1	0.10^*^	177 (139, 241)
16FFa	8.5 ± 3.9	4.5 ± 2.1	6.5 ± 2.5	0.72 ± 0.2^*^	0.74 ± 0.1	0.02	80 (68, 96)
16FFb	9.5 ± 4.3	4.8 ± 2.1	6.8 ± 2.5	0.72 ± 0.1^*^	0.76 ± 0.1	0.04	151 (126, 188)
*Overall*	15.2 ± 7.8	6.5 ± 4.1	7.3^b^	0.74 ± 0.1^b^	0.79 ± 0.1^b^		164 (155, 173)

^*^HWE population deviation and *F*_IS_ at *p *< 0.05; the superscript without same letters indicates statistical significance at *p *< 0.05 among population groups.

Effective population size (*N*_E_) of the populations with a sample size smaller than 30 individuals were not analysed.

### Genetic differentiation among populations

The power of microsatellite markers was high in detection significant population differentiation, where simulations in POWSIM suggested a probability of 100% (χ^2^, Fisher’s test) of detecting a true differentiation of *F*_ST_ = 0.005 under different scenarios of effective population size (Ne) and numbers of generations of drift (t) with 1000 replications (Supplementary Table S4). The mean polymorphic information content of 0.78 was recorded for analysed loci.

The global *F*_ST_ across all 27 populations was 0.022 (*p* < 0.0001), supporting a moderate level of differentiation among individual populations. The highest global *F*_ST_ value of 0.041 was recorded within farmed populations in contrast to the lower levels of global *F*_ST_ observed within the other three population groups: 0.015, 0.010 and 0.009 for the wild, farmed-associated and wild YOY group, respectively. Pairwise *F*_ST_ values among populations are detailed in the Supplementary Table S5. In addition, 268 of 351 pairwise *F*_ST_ comparisons were statistically significant when permuted by Fisher’s exact test. Historical wild samples from 2009 showed high and significant pair-wise differentiation in relation to all populations included in the dataset. The majority of non-significant comparisons were found within adjacent temporal wild populations (2015, 2016) sampled through the Adriatic where *F*_ST_ ranged from −0.002 to 0.005. On the contrary, adjacent temporal farm-associated populations from 2015 and 2016 showed a biannual break in gene flow toward the wild populations, except for recently sampled ones from 2017 (17AB, 17AK) that also showed no significant pair-wise differentiations with farmed populations of Adriatic origin. Interestingly, farm-associated populations paired with the YOY samples from Adriatic nurseries for both sampled years (−0.002 < *F*_ST_ < 0.009). Only the YOY group sampled in the area under aquaculture impact (16JB) showed gene flow towards France-origin farmed fish (15FF). All farmed populations from different origins showed significant pair-wise differentiations within the group (0.009 < *F*_ST_ < 0.076) and between the rest of populations.

**Figure 2:**
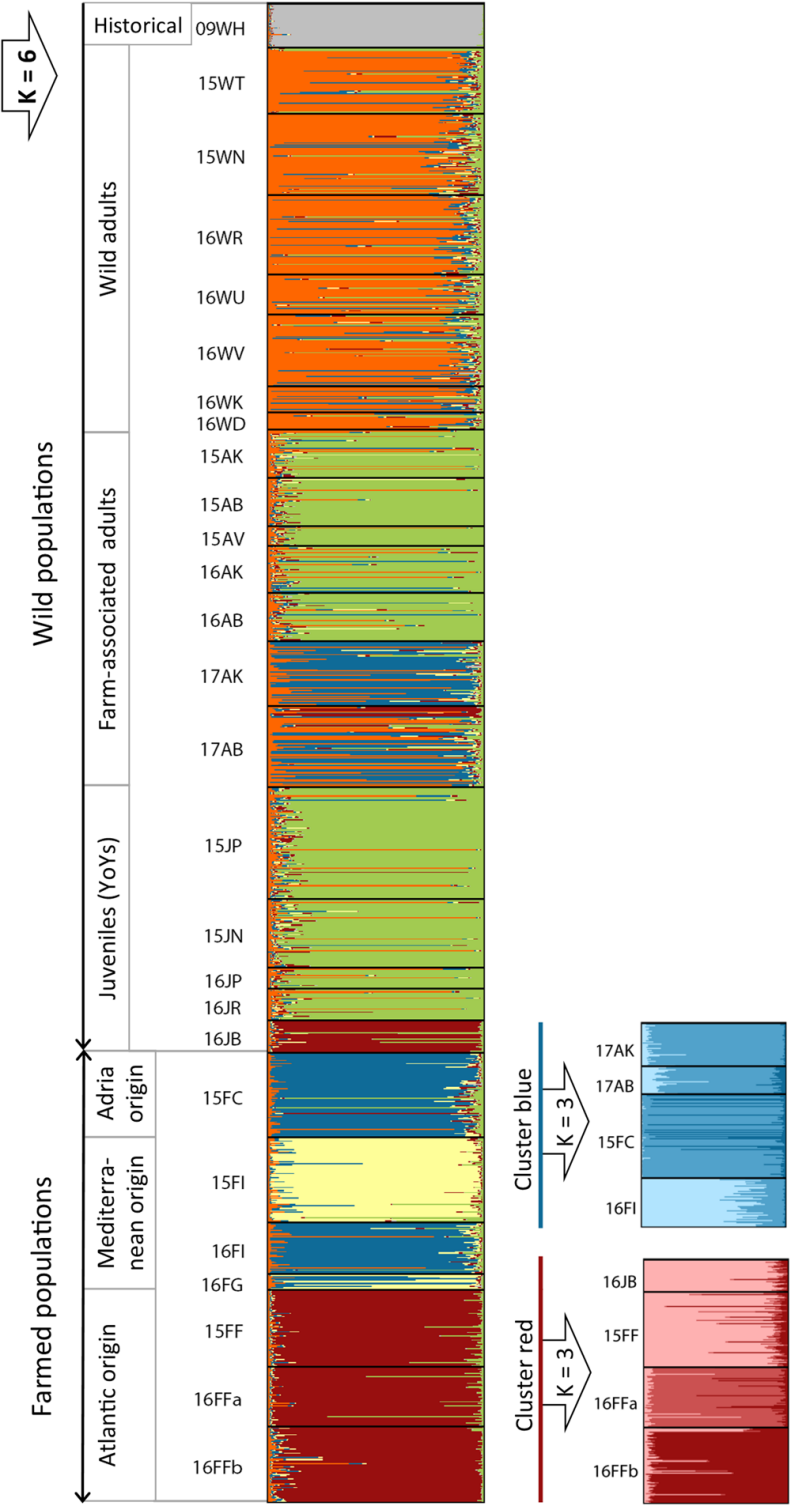
Hierarchical population structure based on Structure analyses of gilthead seabream genotypes at 19 neutral microsatellite loci. The first hierarchical partitioning divided the dataset into six clusters (K = 6). In the second partitioning step, only clusters that showed additional subdivision are presented. Vertical coloured lines represent individual admixture coefficients. Black horizontal lines divide individuals from different sampling sites. Population abbreviations are as in Table 1.

Initial Structure analysis of the dataset suggested K = 6 after application of the Evanno *et al*.^31^ approach. Wild populations sampled within the Adriatic Sea were assigned to five different clusters, of which three clusters were exclusively associated with wild fish origin and two were associated with farmed origins (Fig. 2). Wild historical samples from 2009 formed the first cluster (grey), while recent temporal replicates of wild adults formed the second cluster (orange). In accordance with *F*_ST_ pair-wise results, no gene barrier was observed between 2015 and 2016 temporal replicates of farm-associated adults and temporal replicates of YoYs sampled from different nursery grounds, grouping all populations into the third cluster (green). The YoY population sampled in the area near the finfish aquaculture site (16JB) was assigned to the fourth cluster (red), which also comprised farmed populations of Atlantic origin. Farm-associated adults from 2017 appeared to be admixed with individuals assigned partially to the third wild cluster (orange) and also to the fifth cluster (blue) that comprise farmed populations of Mediterranean origin. Farmed populations originating from western Adriatic broodstock (Italy) formed the separate sixth cluster (yellow).

**Figure 3:**
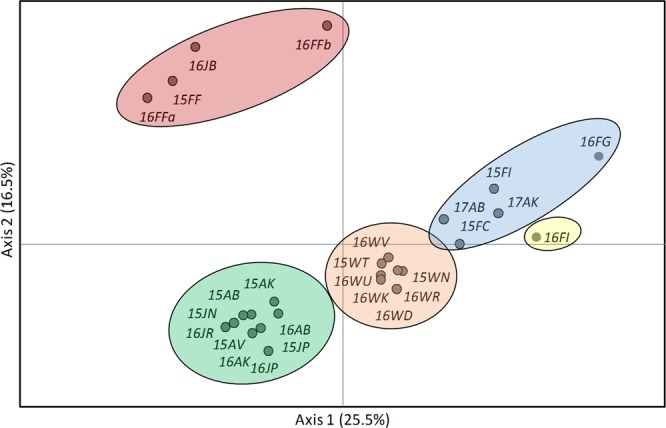
Principal Component Analysis of wild and farmed gilthead seabream populations based on neutral microsatellite allele frequencies. Population groups are indicated in the coloured circles corresponding to their cluster membership identified by Structure (see Fig. 2). Population abbreviations are as in Table 1. Historical wild samples (09HW) were excluded from the analysis.

While no further structure was detected within the adjacent temporal wild populations (orange cluster) and within the adjacent temporal farm-associated populations and YoYs (green cluster), subsequent analysis of each of the cluster of farmed origin (red and blue) resulted in further structure in both subsets, with K = 3 for the Atlantic origin subset, grouping 16JB and 15FF populations into one cluster, and K = 3 for the Mediterranean farmed origin subset with included farm-associated adults 2017, grouping 17AK and 17AB with the eastern Adriatic broodstock (Croatia) (Fig. 2). No further substructure was detected, resulting in a total of K = 10 overall, with clear conformation of farmed origin genotypes in wild populations. These relationships between sampled populations, based on pairwise Nei’s genetic distance, are depicted in the principal coordinates analysis (Fig. 3). An AMOVA arrangement based on the initial Structure analysis when K = 6 revealed that 1.9% of the total genetic variation is explained by differences between major clusters, with evident gene flow among populations within groups (0.8%). Within populations variance accounted for 97.3%. DAPC similarly identified a hierarchical structure in the gilthead seabream dataset and clustered groups following the defined structure observed in Structure software. In successive K-means clustering, the elbow curve of Bayesian Information Criterion (BIC) values indicated the optimal number of clusters of K = 10 (Supplementary Fig. S1).

In respect to the high genetic similarity of YoYs and farm-associated populations from 2015 and 2016, using the 95% critical LOD value given by CERVUS, a total of 6 parent–offspring pairs were identified, connecting YoYs sampled from coastal nursery areas (Neretva, Raša and Pantana) with the parents sampled in the close vicinity of tuna farms. Moreover, 3 YOYs sampled in 2015 from the Neretva River were assigned to the parents sampled around the Kali tuna farm on Ugljan Island (15AK), 2 from the Raša River (2016) and one from Pantana (2015) were assigned to the parents sampled around the Brač tuna farm (15AB), respectively. No parent–offspring pairs were identified among parents from wild populations and YoYs.

### Loci detection power and hybrid identification

Analysis of three simulated data sets, each having a different percentage of hybrids (15%, 33% and 66%), indicated 0.85 and 0.90 as the best thresholds to be applied to assign individuals as non-admixed or admixed in an empirical dataset, balancing among efficiency and accuracy of measurements for testing overall performance of the software Structure in hybrid detection (Supplementary Table S6). The assignment efficiency for all genotype classes was highest in the set with 10% hybrids, except for backcross classes due to a higher overlap with the parental classes that decreased with increasing hybrids in the data set (Supplementary Fig. S2). Still, backcrossed individuals decreased both the accuracy and the efficiency of hybrid detection, as they were difficult to detect, affecting the levels of recommended thresholds (Supplementary Table S6, Hybrid all *vs* Hybrid F1 F2). In comparing the Structure and NewHybrids results, a relatively similar pattern in individual assignment was observed, except for the class of 66% hybrids where high misclassification of purebred individuals as hybrids decreased the overall performance of NewHybrids.

By analysing the real dataset, the Structure results with a threshold q = 0.90 indicated that a total of 75% wild individuals belonged to one of the two recognized clusters with exclusively wild fish origin. Reciprocal hybridization of individuals from wild clusters (orange and green) was noted, where 14% of wild adults and 17.4% farm-associated and YoYs populations showed to be mutually admixed. Moreover, 2.5% individuals from the green cluster were assigned as non-admixed ones to the orange cluster, and vice versa of 4.5% individuals (Fig. 2).

Among 25% of wild individuals that were not assigned to pure wild origin, 10% displayed pure aquaculture ancestry and 15% displayed hybrid-aquaculture ancestry, including hybrids of different farmed origins (Atlantic, Eastern and Western Adriatic) (Table 3). The assignment scores were similar to those obtained by NewHybrids using a threshold q > 0.5, where all hybrids were assigned to the F1 genotype class (Table 3). Different proportions of hybrids were observed in wild populations, with frequencies ranging from 0 to 65%. In general, the smallest hybrid frequencies were recorded in YoY populations (3–13%), whereas the largest frequencies of hybrids were observed in the most recently sampled wild populations in areas impacted by tuna farms (17AK, 65%; 17AB, 37%). Moreover, all juveniles from the YoY group (16JB) sampled within the concession of the Brač farm were assigned to Atlantic farm origin (86.7%) or were identified as hybrids (13.3%). Individuals of pure farm origin, and likely recent escapees, were also found in farm-associated populations (17AK and 17AB), and in wild adult populations (16WR, 16WV and 16WK), with frequencies ranging from 6 to 43% (Table 3).

**Table 3:** Percentage of hybrids with aquaculture ancestry or fish of farmed origin within each wild population identified with the program Structure and NewHybrids.

Wild pop.	Origin	Structure								NewHybrids	
		Hybrids with aquaculture ancestry (%) 0.1 < q < 0.9				Farmed origin (%) q > 0.9				q > 0.5	
		Eastern Adriatic farmed origin	Western Adriatic farmed origin	Atlantic farmed origin	Total/population	Eastern Adriatic farmed origin	Western Adriatic farmed origin	Atlantic farmed origin	Total/population	F1 hybrids (%)	Farmed origin (%)
15WT	W	11.5	—	—	11.5	—	—	—	—	13.1	—
15WN	W	9.2	2.6	3.9	15.7	1.3	—	—	1.3	15.7	2.9
16WR	W	4.0	2.7	—	6.7	6.7	—	—	6.7	27.8	5.6
16WU	W	8.1	—	—	8.1	2.7	—	—	2.7	25.7	2.9
16WV	W	7.4	3.0	1.5	11.9	5.9	—	—	5.9	15.4	4.6
16WK	W	12.5	4.2	8.3	25.0	16.7	—	—	16.7	25.0	12.5
16WD	W	12.5	6.3	6.2	25.0	—	—	—	—	40.0	—
15AK	WA	6.6	—	—	6.6	—	2.2	—	2.2	2.1	—
15AB	WA	—	2.2	—	2.2	—	—	—	—	2.7	—
15AV	WA	—	—	—	—	—	—	—	—	—	—
16AK	WA	4.5	—	2.3	6.8	2.2	—	—	2.2	7.3	—
16AB	WA	2.2	6.7	—	8.9	2.2	—	—	2.2	2.4	4.9
17AK	WA	48.3	10.0	6.7	65.0	43.3	—	—	43.3	50.9	30.2
17AB	WA	22.7	1.3	13.3	37.3	22.6	—	6.7	29.3	40.4	15.8
15JP	YoY	2.9	—	0.9	3.8	—	—	—	—	1.9	—
15JN	YoY	3.1	1.6	3.1	7.8	—	—	—	—	4.8	—
16JP	YoY	5.0	—	—	5.0	—	—	—	—	—	—
16JR	YoY	—	—	3.4	3.4	—	—	—	—	—	—
16JB	YoY	—	3.3	10.0	13.3	—	—	86.6	86.6	—	100
Overall hybrids/farmed					14.8				9.9	14.9	8.0

The impact of farmed origin in each population is listed according to fish membership in the three core farmed-population clusters in Structure.

Pop, populations; q, membership proportion, W, wild adults, WA, wild adults associated with aquaculture sites; YoYs, wild young-of-the-year.

### Connectivity of spawning and nursery areas

**Figure 4:**
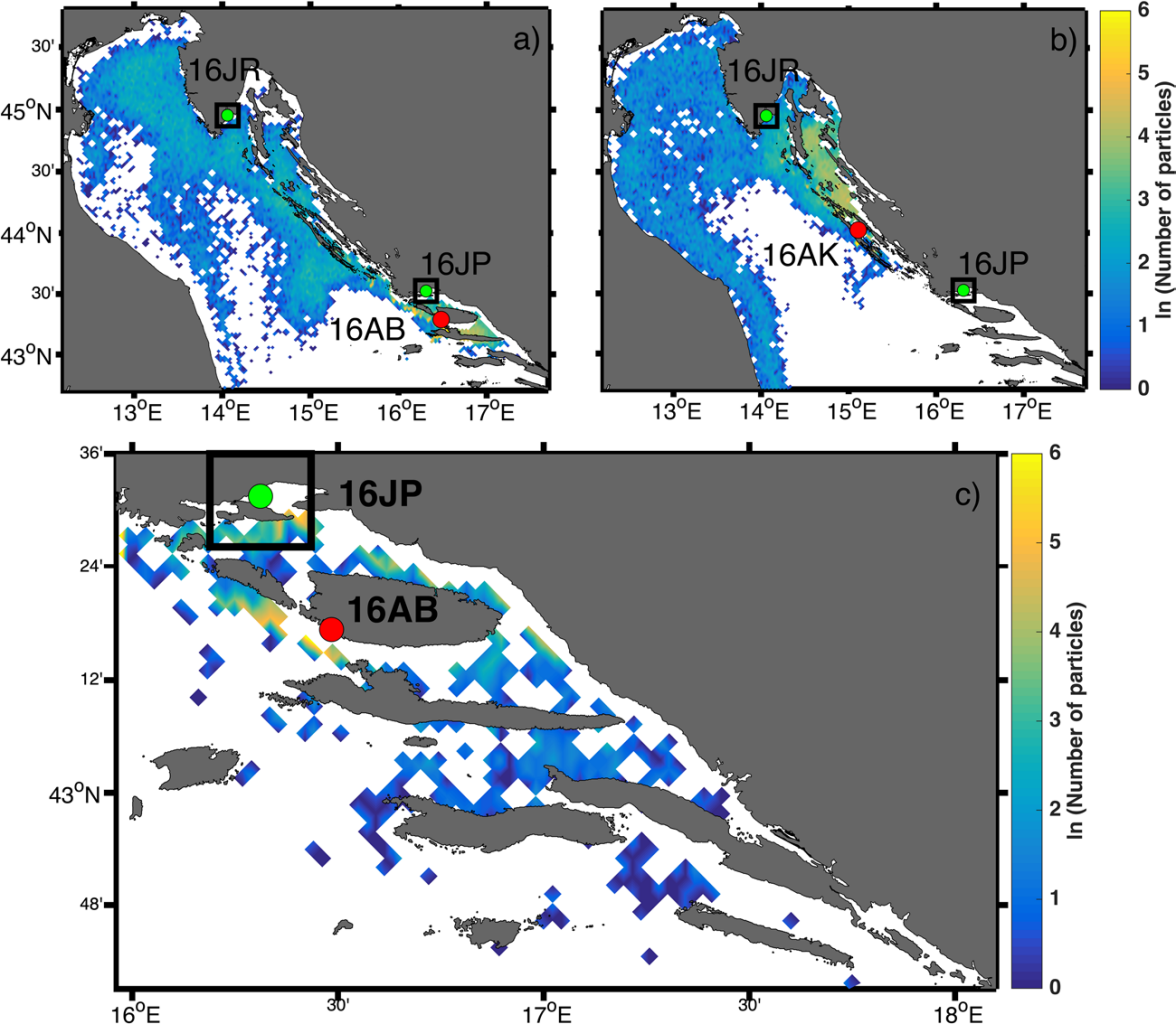
Upper: Spatial particle distribution on 15 May 2016 obtained in the Ichthyop – Adriatic ROMS simulation from the sourced tuna-aquaculture surroundings near islands of (**a**) Brač and (**b**) Ugljan. Source populations are marked with red dots (16AB – Brač, 16AK – Ugljan). The black squares, representing areas of success, are depicted around natural nursery grounds (green dots) where YoY were sampled (16JR – Raša; 16JP – Pantana). Lower (**c**): Spatial particle distributions on 15 May 2016 obtained in the Ichthyop – ASHELF2 ROMS simulation from the sourced tuna-aquaculture surrounding near the island of Brač. The source population is marked with a red dot (16AB – Brač). The black square, representing the area of success, is depicted around a natural nursery ground (green dot point) (16JP – Pantana). The figure has been created using MATLAB 2014a (www.mathworks.com) and GIMP 2.8.16 (www.gimp.org) software.

Spatial particle distributions on 15 May 2016 obtained in the Ichthyop – Adriatic ROMS simulation showed that there was hydrodynamic connectivity between the aquaculture area near Brač Island, as a potential spawning ground for wild gilthead sea breams, and natural nursery ground areas near Pantana and Raša River (Fig. 4a). The same model setup showed connectivity between Ugljan Island and the Raša River (Fig. 4b). The Eastern Adriatic Current (EAC), which is prominent and stronger in southern and central parts of the basin during winter, spring and autumn, transported particles from Brač and Ugljan Islands to Raša River (see Supplementary Methods for details). The Ichthyop – ASHELF2 ROMS simulation for the same date clearly showed connectivity between the aquaculture area near Brač Island and Pantana as a natural nursery ground (Fig. 4c). Averaged modelled current fields produced by the ASHELF2 ROMS simulation indicate interplay between oppositely directed currents (see Supplementary Methods for details). Due to the impact of different wind forces during the first five and half months of 2016 superimposed on thermohaline flow, current direction varied between atypical alignment with the wind and typical inflow direction^32^.

## Discussion

Significant increase of wild gilthead seabream populations in coastal areas of the Mediterranean Sea and its northward expansion has been documented recently, affecting the structure and productivity of ecosystems^3,5,23^. In this study, 19 neutral microsatellites loci were used to explore geographically fine-scale population processes of gilthead seabream within a short-temporal window in coastal areas of the eastern Adriatic Sea, to gain a deeper understanding of the factors shaping genetic connectivity and structure, in comparing 1586 sampled individuals grouped by fish origin (wild *vs* farmed *vs* farmed-associated) and ontogenetic state (juveniles *vs* adults).

The standardized panel of two microsatellites multiplex PCRs for gilthead seabream^30^ applied in this study showed robustness and effectiveness for pedigree analysis and characterisation of populations. Allelic polymorphisms and expected heterozygosities were comparable with those observed in wild and farmed populations of Atlantic and Mediterranean origin^33,34^ and were slightly increased in comparison to previous studies in the eastern Adriatic^4,9^ where only ten loci were used.

The majority of farmed populations showed departure from HWE, due likely to non-random mating and population mixing^35–37^. Similar reports are known^4,9^, including HWE departure of farmed seabass in the Adriatic^24^. Adding wild fish into farmed stocks and swapping breeders between hatcheries is common practice^38^, particularly in case of gilthead seabream where wild males are continuously introduced into aquaculture since most individuals turn into females after their second year of reproduction^39^. Still, lower than average number of alleles per locus, significantly lower allelic richness, heterozygosity and effective size in cultured versus wild populations was recorded within the Adriatic (Table 2). Drops in genetic diversity as a reflection of small effective breeding population size occurs in hatcheries due to high variance in family size and fewer males than females contributing to each mass spawning event^35^. Even though estimates of contemporaneous Ne were significantly smaller in farmed populations than in other wild groups, the majority of farmed populations had F_IS_ values close to zero, suggesting that genetic breeding programmes are implemented in commercial hatcheries of Mediterranean countries in order to control the risk of inbreeding^40^. The heterozygosity deficit and significant departures from HWE observed in Adriatic wild populations at some loci but not systematically for each location can be explained due to the temporary Wahlund effect^41^ caused by several breeding subunits in a sample^4,9,36^ or due to selection. Namely, human-mediated selection due to fisheries may induce evolution toward slow growth, early maturation at small size and higher reproductive investment where the latter two characteristics are already observed in certain gilthead seabream populations from eastern Adriatic^7^.

Even though gilthead seabream is an important commercial species for the aquaculture and fisheries sectors, population genetic characteristics throughout the range of species distribution are still not clearly resolved. Different types of markers produce incoherent and puzzling patterns of gene flow discontinuity among regions. Some authors indicated either a weak population structure in the Atlantic and Mediterranean Sea^42–45^ or a strong genetic subdivision at short distances along the basins^9,46^. On the other hand, farmed fish generally form genetically distinct groups compared to their proximal wild counterparts^36,37,42^. This pattern is confirmed in the present study where the main source of genetic differentiation was found among farmed populations of various origins (*F*_ST_ = 0.041) and between farmed and wild populations, except for interactions with wild populations (17AB, 17AK, 16JB) in which a high number of escapees/hybrids were recorded. A similar pattern of inter- and intra-farmed strain divergence, as a result of the selection in breeding programs, the founder effect and genetic drift, was observed for other farmed species^24,47^.

Another source of genetic divergence was found among wild genotype collections (*F*_ST_ = 0.012). While wild adults from natural locations showed homogenous genetic pattern along the Croatian coast, wild adults sampled around tuna farms together with wild young-of-the-years sampled from three coastal nursery grounds showed multiannual genetic connectivity and a clear break in gene flow toward the wild adults from natural locations. At a small spatial scale, such gene flow discontinuity observed among wild fish collections adds new insight into population structure dynamic, considering that previous studies using fewer loci indicated a lack of any population subdivision within the Adriatic^9,33^. Low values of *F*_ST_ and high gene flow in marine species do not necessary imply an absence of population structure^48,49^ and the observed differences could be due to the different resolution of molecular markers used.

The extent of movements of adult *S. aurata* is still little known^50^, though the long pelagic larval duration up to 50 days at 17–18 °C is likely to contribute to the exchange of migrants among populations^51^. Considering that wild adults that appear in large school formations during late autumn and winter showed genetic divergence from wild offspring in temporal replicates, it could be argued that those populations function as independent units and conduct an exclusively trophic north-to-south migration along the eastern Adriatic coastline, and spawning occurs elsewhere. This is further supported by the parental assignment analysis where no parent–offspring pairs were identified. Thus, we can characterize wild gilthead seabream populations from Croatian waters into two contingents with non-overlapping spawning grounds, (i) migratory and (ii) residential adults, i.e. ones observed around tuna farms. Due to the high dispersal capacity of the studied species, these two contingents cannot be considered as completely isolated units, since the Structure analysis identified a bidirectional pattern of hybridization at lower frequencies (<17%). In Croatian waters, migratory adults first appear during autumn in the north-eastern parts and subsequently move southwards, and therefore, we can speculate that they originate from Italian natural lagoons, i.e. immigrant donor areas that are very important nurseries and feeding grounds for seabream along the western coast^52^. However, more sampling is required along the entire Adriatic coast to obtain a representative pattern of genetic connectivity.

Estimates of effective population size recorded from residential adults suggest the existence of large breeding populations, which is in line with several recent studies that portrayed Adriatic tuna farms areas as a permanent residency of highly abundant wild seabream^6,7^. The aggregative behaviour has been primarily attributed to the permanent and increased availability of tuna baitfish losses, composed of small pelagic species rich in omega-3 and 6 fatty acids. In addition to the observed morphological differences, farm-associated seabream had a higher reproductive potential than other wild populations, due to the fish-enriched diet^7^. It seems that reliance on this trophic source has a positive influence on successful spawning and development of fish egg and larvae^53^ considering the strong genetic connectivity observed between resident adults from three tuna-farm locations with offspring collections in the three coastal nursery grounds, suggesting these sites are spawning areas. This is further supported by parental test analysis where six parent–offspring pairs were identified, connecting parents from the farm surroundings of Brač and Ugljan Islands with offspring from all three nurseries (Raša, Pantana and Neretva) situated along the coastline. In addition, the coupled numerical system with ROMS and Ichthyop models successfully utilized in tuna offspring distribution studies within the Adriatic^54^, has confirmed a possible passive transport between tuna-farms areas off the islands of Brač and Ugljan and the Raša and Pantana nursery grounds in 2016.

Both the multivariate and Bayesian based clustering analysis identified similar structure architecture within the basin, supporting the populations *F*_ST_ pair-wise estimates. Hierarchical Structure and DEPC analysis gave 10 distinct clusters, three of which were exclusively associated with the wild fish origin and seven with farmed ones, where each unit corresponded to a different hatchery source of fry. Namely, due to the limited number of national hatcheries, the majority of fry is still imported from France, Italy and Greece. While all historical samples were completely assigned to a single cluster, several wild populations showed to be heavily admixed with different farmed origins. This is especially true for the recently sampled populations around both tuna farms (17AK, 17AB) where a complete shift in population structure was recorded in 2017 in comparison to 2015 and 2016. Both tuna-farms are located few kilometres from seabream-cages farms, and it is possible that seabream escapees from neighbouring farms were attracted by tuna waste fish feed at the time of fish sampling. Namely, the presence of other farms has influenced the movement patterns of escaped fish, by moving repeatedly from the release cages to other adjacent farms^55,56^. The overall number of escapes detected in the wild samples was close to 10%, which is consistent with previous studies^9,24,57^; however, more than half of the escapees in this study were captured at the aquaculture sites. Moreover, offspring 16JB collected from the natural bay near a seabream farm in middle Adriatic showed pure Atlantic-farmed origin, and they clustered together with farmed adults sampled in 2015 from the same area, suggesting that they may have originated from eggs spawned by farmed fish inside the cage. Escape through spawning has already been recognized in Greece and authors suggested that this mechanism may be responsible for recruitment peaks in sea bream populations in certain coastal areas of Greece^5^. According to the risk assessment of the environmental impact of farming^58^, the observed number of escapees (<10%) in this study presents a moderate risk for further genetic changes in wild populations. Interestingly, genetic temporal changes in population structure has already been recorded here, considering that the wild historical samples collected during an earlier stage of farming (in 2009) showed high genetic divergence toward wild contemporary samples. Long-term and cumulative introgression of farmed fish and the density of the native populations have been recognized as the main drivers of the temporal genetic change in wild salmonids populations^25,26^.

Overall performance of simulated hybrid genotypes using both Structure and NewHybrids indicated that our dataset provided the necessary resolution to identify recent hybrids, but failed to distinguish backcross hybrids with high efficiency, as also seen elsewhere^24,59,60^.

About 15% of the hybrids in this study were found to have an aquaculture ancestry, with a similar non-random spatial distribution to those of escapees, demonstrating successful genetic hybridization and introgression of farmed escaped gilthead seabream in recipient wild populations. These wild populations showed slightly reduced allelic diversity (16WK, 16WD) or reduced Ne (17AK) in comparison to non-admixed ones. In the long run, repeated escapes of farmed fish in a wild population may cause a decline in the fitness of the recipient population due to reduced genetic variability that might affect the ability of native populations to cope with a changing environment^61,62^.

Studies of farmed seabream introgression in other regions are scarce in contrast to the extensive work conducted in Norway salmon rivers^25,26,63–65^. The latest research^27^ indicates significant introgression in half of the wild populations studied, with levels of introgression in excess of 10% in 27 of 109 rivers. Overall, the authors reported decreased genetic differentiation among populations over time^25–27^. Similar patterns of introgression level and temporal population instability have been recorded here. Due to the high larval dispersal potential and large natural variation in fish survival, impacted also by climate change and other anthropogenic factors, the degree to which these processes contribute in shaping contemporary regional population genetic patterns is more challenging than introgression estimation per se. Despite the continuous introgression of farmed seabream in the past decade^4,9^ where wild populations near aquaculture facilities were most affected, it should be noted that this warm-water species shows a highly adaptive behavioural response and evolutionary capacity to prevailing conditions by increasing its dominance in coastal ecosystems and capacity for range changes towards the northern edge of its distribution. While the solid genetic base of wild and farmed strains from the eastern Adriatic is established here, as a first step toward development of mitigation strategies to prevent further erosion of genetic integrity, abovementioned adaptations require further attention.

## Material and Methods

### Ethics statement

The methods involving animals in this study were conducted in accordance with the Laboratory Animal Management Principles of Croatia. All experimental protocols were approved by the Ethics Committee of the Institute of Oceanography and Fisheries.

### Fish sampling

To obtain fine population structure resolution in the eastern Adriatic Sea, a total of 1586 individuals of gilthead seabream were collected from 22 different sampling sites between July 2015 and January 2017 (Table 1; Fig. 1). Collection included (i) wild adults (2 or 3 years of age, 16–22 cm total length) sampled from seven natural locations in two consecutive years (2015, 2016), covering 600 km of Croatian coastline; (ii) farm associated adults sampled from the two main aquaculture areas (Brač and Ugljan Islands) impacted by combined tuna and seabream farms in three consecutive years (2015, 2016, 2017); (iii) wild young-of-the-year (YoY) with total length ranging from 2.9 to 4.5 cm sampled in the natural nursery grounds of three coastal rivers mouths (Raša, Pantan and Neretva) and one bay located in the vicinity of fish farm in two consecutive years (2015, 2016), and (iv) farmed fish of different origin, i.e. imported Italian fingerlings originating from western Adriatic broodstock, French fingerlings originating from various Atlantic broodstocks, Greeks fingerlings and domestic hatchery fingerlings from the eastern Adriatic broodstock sampled during 2015 and 2016 (Table 1). All farms included in this study are active and annually produce more than 100 tons of fish. In addition, historical samples of wild gilthead seabream from Šegvić-Bubić *et al*.^9^, collected from the Brač Channel in 2009, were included in the analysis to test temporal stability in allele frequencies. Fish sampling was conducted in collaboration with local fishermen and farmers. Fish were measured and weighted, and the distal portion of fin-clips was clipped and stored in 96% ethanol for subsequent genetic analysis. Depending on location, sample sizes ranges from 19 to 124 individuals (Table 1).

### Microsatellite genotyping

Total genomic DNA from fins was extracted by proteinase K digestion, followed by a simplified DNA isolation procedure^66^. DNA quality and quantity were assessed by spectrophotometry (IMPLEN N50, Germany), and each sample was diluted to 15 ng μL^−1^ in DNAse/RNAse free water. Total data set containing 1586 individuals from 20 locations was characterized using two multiplex PCRs, SMsa-1 and SMsa2 (SuperMultiplex *Sparus aurata*), developed by Lee-Montero *et al*.^30^ with 21 specific microsatellite markers applied (Supplementary Table S1). Using the Multiplex PCR kit (Qiagen, Germany), amplification of the loci was run in 10 μL reactions containing 15 ng DNA on an Eppendorf Mastercycler Nexus Gx2 thermal cycler, having final concentrations of all primers uniformly set at 0.2 μM. For both multiplex PCRs, conditions were as follows: initial denaturation at 95 °C for 5 min, 26 cycles at 95 °C for 30 s, annealing at 60 °C for 90 s, and elongation at 72 °C for 30 s; and then a final elongation of 30 min at 60 °C. Fragments were separated on an ABI3130 automated sequencer (Applied Biosystems) while electropherograms and genotypes were analysed using GeneMapper software v.3.5 (Applied Biosystems).

### Hardy-Weinberg equilibrium, linkage disequilibrium and null alleles

MICROCHECKER 2.2.3 software^67^ was used to identify possible genotyping errors such as stuttering, large allele dropout and null alleles, while FreeNA^68^ was used to check the likelihood of null alleles present following the Expectation Maximization (EM) algorithm. The bootstrap 95% confidence intervals (CI) for the global *F*_ST_ values were calculated using 50,000 replicates over loci. Tests for linkage disequilibrium (LD) and deviations from Hardy-Weinberg equilibrium (HWE) for each locus and population were estimated using unbiased exact tests and Markov Chain algorithm (10,000 dememorization steps, 100 batches and 5000 iterations) implemented in GENEPOP v.3.1b^69^. The significance of HWE and LD values were adjusted using the sequential Bonferroni correction for multiple pairwise testing^70^.

### Genetic diversity and effective population size

The number of alleles (N) and mean effective number of alleles across loci (Ae) were calculated using POPGENE v.1.32^71^, while the inbreeding coefficient (*F*_IS_) and allelic richness (Ar) were calculated using FSTAT v.2.3^72^. ARLEQUIN v.3.5^73^, was used to calculate observed (Ho) and expected (He) heterozygosity. Contemporary effective population size (*N*e) was estimated in the program NeEstimator V2^74^ using the single-sample linkage disequilibrium method for populations with a sample size over 30 individuals. Low frequency alleles ≤0.02 were excluded from the analysis to minimize potential bias caused by rare alleles. Using putatively neutral loci, parent–offspring matches were analysed using the software CERVUS^75^. The genotyping error rate was set to 1%, and three different estimates (0.01, 0.1 and 0.2) of the proportions of candidate parents sampled were evaluated. The critical LOD value was set at strict 95% confidence, where final LOD value cut-off for identifying parent–offspring pairs was always above 2. The tested dataset included allele frequencies of wild, farm-associated YOY populations.

### Genetic differentiation and population structuring

POWSIM^76^ was used to assess the statistical power of tests for genetic homogeneity on the applied set of markers and sample sizes. Each simulation was run 1000 times and power was determined as the proportion of simulations that Fisher’s exact test detected as significant at the 0.05 level. Pair-wise and global *F*_ST_ values calculation were performed in ARLEQUIN v.3.5^73^, where confidence levels were estimated by 1000 permutations of the datasets.

To infer genetic structure in the neutral microsatellite dataset and the extent of admixture between wild and farmed individuals, different statistical tests were performed, including multivariate and Bayesian based clustering analysis. Based on pairwise Nei’s genetic distance matrix, a principal coordinates analysis (PCoA) was conducted using the GENALEX 6. Software^77^. Discriminant Analysis of Principal Components (DAPC)^78^ from *adegenet*v1.4-1^79^ in R was used to further characterize the potential differences between historical, present-day wild, and farmed fish. The function *find.clusters* and ‘*k*-means’ algorithm determined the most likely number of genetic clusters in the data and function *xvalDapc* was used sequentially using 1000 replicates, to determine the optimal number of principal components to retain in the discriminant analysis.

In addition to nonparametric analysis, ParallelStructure^80^ an R-based implementation of the program Structure^28^ on the CIPRES portal^81^ was used to identify genetic clusters and to evaluate the extent of admixture among them. An admixture model was used to determine the number of population clusters (K) with a burn-in of 200,000 followed by 1,000,000 iterations with default parameters and no prior structuring information. *K* values from 1 to the maximal number of sampled groups were assessed, with 10 replicates per *K* value. The number of genetically homologous clusters (if greater than two) was determined using the ad hoc statistics ΔK^31^ implemented in STRUCTURE HARVESTER^82^ and visualized using CLUMPP^83^ and DISTRUCT^84^. To determine the presence of hierarchical structuring of population where one genetic group can be made of several subgroups, the above procedure was repeated in successive steps for all subgroups identified with the deltaK or lnP (D) method until the lowest level of differentiation was recorded in all subgroups or when a subgroup corresponded to a single locality. Analysis of molecular variance (AMOVA) was conducted with Arlequin 3.5. using 20000 permutations on population groupings identified by the Structure analyses.

### Hybrid detection

The optimal threshold values to distinguish an individual as admixed in the Structure analyses, or to assign it to a particular hybrid class in NewHybrids^85^, were identified following the approach of Vähä and Primmer (2006)^86^. In brief, we first selected 50 pure wild and pure farmed individuals whose Q-values were >0.95 in the preliminary Structure analysis using the neutral microsatellites panel. Next, we created simulated data sets of each of six genotype classes (pure parents, first and second-generation hybrids and F1 backcross with a pure parent) using the R packages hybriddetective^87^. To compare the overall performance of each software for hybrid detection, three sets containing different proportions of hybrids (15%, 33%, 66%) were constructed and analysed separately. The simulated data sets were then run through Structure using k = 2 and the above settings for the real dataset, and NewHybrids to determine the efficacy (the proportion of individuals in a group that were correctly identified), accuracy (the proportion of an identified group that truly belongs to that category) and performance (the product of efficiency and accuracy) of these programs in detecting hybrid individuals. For NewHybrids, we used a posterior probability of 50% as the threshold, while for Structure we used threshold values ranging from 0.60 to 0.95 to analyse the assignment of an individual to a specific class. Finally, the threshold value having the most suitable performance was later used to analyse the hybrid percentage in the real dataset.

We implemented NewHybrids v.1.1^85^, which provides the posterior probability that an individual belongs to one of six possible classes that differ in the extent of admixture, in our case farm, wild and hybrids (F1, F2 and backcrosses), by using Jeffreys prior probabilities and default genotype proportions, following a burn-in period of 20,000 generations and 200,000 MCMC. The simulated pure populations were included in the real population data sets using the ‘z’ and ‘s’ designations indicating pure wild or farmed genotypes.

### Hydrodynamic and Lagrangian modelling

Due to high gene flow observed among tuna farm-associated and YoY populations (see Results), a coupled modelling system with hydrodynamic Regional Ocean Modeling System (ROMS, www.myroms.org)^88–93^ and Lagrangian individual based model Ichthyop v.3.3 (IBM, www.ichthyop.org)^94–97^ was set up to infer larval supply in 2016 of the gilthead seabream adults aggregated around two main tuna aquaculture areas (16AB – Brač Island and 16AK – Ugljan Island) with the natural nursery ground areas (16JR – Raša; 16JP – Pantana; Fig. 1) and to test the correlation between genetic and transport connectivity. Two different ROMS setups were used to calculate input fields for IBM (hourly averaged current, temperature and salinity fields) for the period from 1 January, as seabream spawning regularly occurs during first two months of the year, to 15 May 2016 when juvenile sampling was conducted. The first ROMS setup (Adriatic ROMS)^54^ covered the entire Adriatic Sea with a resolution of 2.5 km, while the second ROMS setup (ASHELF2 ROMS)^32^ had a smaller domain encompassing the eastern coastal area of the middle Adriatic, with a resolution of 1 km (Fig. 4). Further details of both ROMS applications are provided in the Supplementary Methods. IBM was used to calculate the Lagrangian dispersion of gilthead sea bream plankton stages. During the period from 1 January to 29 February, each day at 17 h, 1000 particles were released from sources at tuna aquaculture areas. Particles were released from stain with a radius of 2 km located at depth between 30 and 60 m (see Stagličić *et al*.^6^ for gilthead sea bream depth distribution). Spatial particle distribution was calculated at the end of the simulation on 15 May 2016, the date of sampling of gilthead sea bream (16JP, 16JR) at nursery locations (see Supplementary Methods). Beaching coastal behaviour was applied throughout the entire domain but with modification suitable for nursery brackish water areas. Namely, outside the area of success, particles that arrived at the coast were neglected in the further dispersion calculations. On the other hand, their arrival at the coast in the area of success around nursery grounds was counted as a successful outcome.

## Supplementary information

Supplementary information

## Data Availability

The full dataset of genotypes has been deposited into GenoBase of Institute of Oceanography and Fisheries (http://jadran.izor.hr/~tsegvic/aquapop/GenoBase.html) and is available from the corresponding authors on reasonable request.
