# Supplementary material for: Spatial connectivity pattern of expanding gilthead seabream populations and its interactions with aquaculture sites: a combined population genetic and physical modelling approach
Source: Sci Rep. 2019 Oct 11;9:14718. doi: 10.1038/s41598-019-51256-z (PMC6788985; doi:10.1038/s41598-019-51256-z)
Supplement: Supplementary file 1 — Supplementary information [file 41598_2019_51256_MOESM1_ESM.docx]

**Supplementary information**

**Spatial connectivity pattern of expanding gilthead seabream populations and its interactions with aquaculture sites: a combined population genetic and physical modelling approach**

Žužul, Iva^1^; Šegvić-Bubić, Tanja*^1^; Talijančić, Igor^1^; Džoić^1^, Tomislav; Lepen-Pleić^1^, Ivana; Beg Paklar, Gordana^1^; Ivatek-Šahdan, Stjepan^2^; Katavić, Ivan^1^; Grubišić, Leon^1^

^1^Institute of Oceanography and Fisheries, PO Box 500, Šetalište Ivana Meštrovića 63, 21000 Split, Croatia

^2^Meteorological and Hydrological Service of Croatia, Grič 3, Zagreb, Croatia

*Corresponding author: [tanja.segvic@izor.hr](mailto:tanja.segvic@izor.hr)

**Supplementary Table S1**. Primer sequences, fluorescent dyes, linkage group and combinations of primers in two (SMsa1, SMsa2) multiplex PCRs, number of alleles, type of nucleotide motif and the references for each microsatellite loci are provided. Loci M3 and H8 were omitted from further analyses. For more details, see the Results section.

| **Multiplex PCR** | **Primer code** | **Locus** | **5’-3’ sequence of the primer** | **5’ fluorescent dye** | **Linkage group** | **Nucleotide motif** | **Allele size range (bp)** | **Number of alleles** | **Reference** |
| --- | --- | --- | --- | --- | --- | --- | --- | --- | --- |
| SMsa1 | E1 | Bd-68-T | F: AGGGGCGTTTCAGGTTCT  R: gtttAGGTGAGTCCGTCAATCAGTGTAT | 6-FAM | 23 | Di | 72–90 | 13 | Lee Montero et al. (2013)^30^ |
|  | C3 | EId-39-T | F: GAACTGTGTGAGAGAGGACAGTTG  R: GAAGGAAAAATTCTGATTGGAGTTC | 6-FAM | 6 | Di | 104–164 | 43 | Lee Montero et al. (2013)^30^ |
|  | D4 | P3 | F: GAGGGGACACGTAAATTTGG  R: gtttAACAACAGTCAAATGCTGAGTTGT | 6-FAM | 1 | Di | 171–207 | 23 | Lee Montero et al. (2013)^30^ |
|  | D11 | CId-29-T | F: GCTCAGCACTACTTTAGTGTTTTGG  R: gttCTGCAGGGAGGAAACAAGAC | VIC | 5 | Di | 76–92 | 14 | Lee Montero et al. (2013)^30^ |
|  | C12 | Dt47 | F: TGCCTCTCTTTCACTCACTTCTC  R: GCACATTGCTCCACACAGAG | VIC | 18 | Di | 121–137 | 10 | Lee Montero et al. (2013)^30^ |
|  | I9 | At37 | F: GGTTAGGGTAATCAGAAATGCAATG  R: gtttCTTCTCCAGTCACGATCAATAAAG | VIC | 19 | Tetra | 142–178 | 11 | Lee Montero et al. (2013)^30^ |
|  | E4 | BId-18-F | F: AGTGATGCGCTCTGGGTTTTA  R: GTCTCTCAGCCTTTGAAGTGTTATC | NED | 11 | Di | 64–92 | 26 | Lee Montero et al. (2013)^30^ |
|  | A5 | Bt-14-F | F: AGCCGAGTACTTCTACTCCTCTGAT  R: gtttAGTGAGGGCGGACAGATAAAG | NED | 20 | Di | 106–112 | 8 | Lee Montero et al. (2013)^30^ |
|  | M5 | Ct27 | F: GAGACAGAGAGGAAGAAAAAGGATT  R: gtttCAATGCTACAAGCTGCCTCAG | NED | 24 | Tetra | 128–232 | 48 | Lee Montero et al. (2013)^30^ |
|  | L11 | DId-16-F | F: GTTGTAGATCGGAGTGTGATAACG  R: gttTGCTCCGAGTAAGCCATATGTA | PET | 10 | Di | 103–113 | 9 | Lee Montero et al. (2013)^30^ |
| SMsa2 | J1 | P96 | F: CGCAATTAGAAGTAGGAGACTGG  R: gttTGCCAGATGCAGGATGTAAG | 6-FAM | 5 | Di | 76–102 | 29 | Lee Montero et al. (2013)^30^ |
|  | G2 | CId-03-F | F: ATGACTAAACATCACGTTCATGGAT  R: gtttAGCTCATGGCTAACTGTGTACTTTT | 6-FAM | 13 | Di | 113–137 | 24 | Lee Montero et al. (2013)^30^ |
|  | G3 | Hd-23-T | F: TCTAACTTTCTTTGAGGTCCCTCTT  R: GAGGACAAGCTGAAGTACGAGTC | 6-FAM | 26 | Di | 150–170 | 12 | Lee Montero et al. (2013)^30^ |
|  | M3 | BId-04-F | F: GATCTCATTATGACGGATCATTAGC  R: gtttATCTTTGTCCGCATGTTTCAC | VIC | 7 | Di | 83–95 | - | Lee Montero et al. (2013)^30^ |
|  | H8 | P60 | F: ATGCTGACATAACACAATGTAGCTC  R: GAGAAGTGAGGGGATACCTGAG | VIC | 19 | Di | 108–116 | - | Lee Montero et al. (2013)^30^ |
|  | I8 | B13b | F: ATGACAGTGTTTGAGCTCAGTGTTA  R: gtttCCTGCATTCCAGCTTCAGAT | VIC | 3 | Di | 128–144 | 33 | Lee Montero et al. (2013)^30^ |
|  | L7 | Dd-57-T | F: ATCATTGTTCAGATAATGGGACAC  R: gtTTCAAAATCTTTTGGTCTGTGC | NED | 16 | Di | 76–80 | 6 | Lee Montero et al. (2013)^30^ |
|  | H5 | Hd-15-H | F: CTCTCTCATGCGCACTTTCTT  R: gtttCACTCCCTCTGATTTATGAGATGAT | NED | 25 | Di | 91–123 | 23 | Lee Montero et al. (2013)^30^ |
|  | B6 | Dt23 | F: ACACAACACACGATTACAGCAGA  R: gttTCCATGTGAGATGTCACTCTATTTC | NED | 20 | Tetra | 140–208 | 22 | Lee Montero et al. (2013)^30^ |
|  | F4 | P54 | F: TGTCTCTCTATTGTCCTCTCTCCTC  R: gttTGCACCTACAGCGGCATC | PET | 14 | Di | 66–90 | 13 | Lee Montero et al. (2013)^30^ |
|  | C10 | EId-38-F | F: TCTGAATAAAACATTGTCTGCAGTG  R: gtttCTCAGACGGAGTATTTTTGGAACA | PET | 23 | Di | 127–161 | 48 | Lee Montero et al. (2013)^30^ |

**Supplementary Table S2.** Summery statistics of 19 neutral microsatellite loci^30^ among samples of gilthead seabream *Sparus aurata*.

| **Lo** |  | **09**  **WH** | **15**  **WT** | **15**  **WN** | **16**  **WR** | **16**  **WU** | **16**  **WV** | **16**  **WK** | **16**  **WD** | **15**  **AK** | **15**  **AB** | **15**  **AJ** | **16**  **AK** | **16**  **AB** | **17**  **AK** | **17**  **AB** | **15**  **JP** | **15**  **JN** | **16**  **JP** | **16**  **JR** | **15**  **JB** | **15**  **FC** | **15**  **FI** | **16**  **FI** | **16**  **FG** | **15**  **FF** | **16**  **FFa** | **16**  **FFb** |
| --- | --- | --- | --- | --- | --- | --- | --- | --- | --- | --- | --- | --- | --- | --- | --- | --- | --- | --- | --- | --- | --- | --- | --- | --- | --- | --- | --- | --- |
| **E1** | **n** | 41 | 80 | 90 | 89 | 47 | 86 | 29 | 19 | 56 | 44 | 21 | 50 | 46 | 60 | 70 | 124 | 71 | 25 | 34 | 30 | 96 | 97 | 57 | 15 | 71 | 59 | 79 |
|  | **nA** | 10 | 10 | 8 | 10 | 9 | 9 | 8 | 8 | 7 | 9 | 8 | 8 | 9 | 9 | 9 | 9 | 9 | 8 | 9 | 4 | 9 | 8 | 7 | 6 | 5 | 5 | 5 |
|  | **HO** | 0.76 | 0.84 | 0.77 | 0.76 | 0.68 | 0.78 | 0.79 | 0.79 | 0.73 | 0.82 | 0.86 | 0.78 | 0.91 | 0.87 | 0.81 | 0.77 | 0.77 | 0.84 | 0.82 | 0.73 | 0.77 | 0.77 | 0.70 | 0.67 | 0.66 | 0.85 | 0.76 |
|  | **HE** | 0.82 | 0.84 | 0.80 | 0.80 | 0.80 | 0.80 | 0.77 | 0.83 | 0.79 | 0.79 | 0.80 | 0.79 | 0.84 | 0.85 | 0.82 | 0.78 | 0.78 | 0.79 | 0.79 | 0.75 | 0.80 | 0.78 | 0.69 | 0.72 | 0.73 | 0.78 | 0.73 |
|  | **FIS** | 0.08 | 0 | 0.04 | 0.04 | 0.16 | 0.03 | -0.05 | 0.10 | 0.08 | -0.04 | -0.07 | 0.01 | -0.09 | -0.02 | 0.01 | 0.02 | 0.01 | -0.06 | -0.04 | 0.02 | 0.04 | 0.01 | -0.01 | 0.08 | 0.09 | -0.09 | -0.04 |
| **C3** | **n** | 40 | 80 | 89 | 89 | 47 | 86 | 29 | 19 | 56 | 44 | 21 | 50 | 46 | 60 | 70 | 124 | 71 | 25 | 34 | 30 | 96 | 97 | 57 | 15 | 71 | 59 | 79 |
|  | **nA** | 27 | 32 | 29 | 27 | 29 | 30 | 21 | 17 | 25 | 29 | 15 | 26 | 26 | 28 | 29 | 36 | 27 | 20 | 19 | 16 | 31 | 24 | 19 | 12 | 22 | 18 | 21 |
|  | **HO** | 0.90 | 0.94 | 0.90 | 0.88 | 0.98 | 0.93 | 0.93 | 0.89 | 0.93 | 0.91 | 0.95 | 0.96 | 0.96 | 0.88 | 0.91 | 0.95 | 0.87 | 0.84 | 0.94 | 0.93 | 0.90 | 0.95 | 0.91 | 1 | 0.92 | 0.95 | 0.85 |
|  | **HE** | 0.95 | 0.95 | 0.96 | 0.95 | 0.95 | 0.95 | 0.95* | 0.94 | 0.94 | 0.95 | 0.93 | 0.96 | 0.96 | 0.96 | 0.96 | 0.95 | 0.94 | 0.95 | 0.94 | 0.9 | 0.94 | 0.82 | 0.94 | 0.91 | 0.93 | 0.90 | 0.92 |
|  | **FIS** | 0.06 | 0.01 | 0.06 | 0.08 | -0.03 | 0.02 | 0.02 | 0.003 | 0.02 | 0.05 | -0.02 | -0.004 | -0.00 | 0.08 | 0.05 | 0.003 | 0.07 | 0.11 | 0.002 | -0.04 | 0.05 | -0.02 | 0.03 | -0.10 | 0.02 | -0.05 | 0.08 |
| **D4** | **n** | 40 | 80 | 88 | 88 | 45 | 83 | 28 | 18 | 56 | 42 | 21 | 50 | 43 | 58 | 64 | 123 | 71 | 24 | 34 | 30 | 96 | 97 | 57 | 15 | 62 | 59 | 72 |
|  | **nA** | 14 | 12 | 14 | 14 | 12 | 14 | 11 | 13 | 13 | 11 | 11 | 15 | 12 | 13 | 16 | 14 | 14 | 11 | 13 | 9 | 13 | 11 | 12 | 7 | 9 | 9 | 9 |
|  | **HO** | 0.6 | 0.68 | 0.74 | 0.74 | 0.64 | 0.71 | 0.82 | 0.78 | 0.64 | 0.62 | 0.67 | 0.64 | 0.7 | 0.71 | 0.75 | 0.73 | 0.65 | 0.75 | 0.62 | 0.77 | 0.67 | 0.71 | 0.67 | 0.73 | 0.63 | 0.66 | 0.69 |
|  | **HE** | 0.83* | 0.78 | 0.85 | 0.82 | 0.85 | 0.86 | 0.84 | 0.84 | 0.79 | 0.77 | 0.81 | 0.84 | 0.87 | 0.83 | 0.87 | 0.84 | 0.83 | 0.80 | 0.88* | 0.83 | 0.79 | 0.82 | 0.74 | 0.74 | 0.83* | 0.81 | 0.84 |
|  | **FIS** | 0.28* | 0.13 | 0.13 | 0.11 | 0.25 | 0.17 | -0.03 | 0.08 | 0.19 | 0.20 | 0.18 | 0.24 | 0.20 | 0.15 | 0.14 | 0.13 | 0.22* | 0.07 | 0.30 | 0.08 | 0.15 | 0.13 | 0.10 | 0.02 | 0.25 | 0.18 | 0.18 |
| **D11** | **n** | 41 | 80 | 90 | 89 | 46 | 86 | 29 | 19 | 56 | 44 | 21 | 50 | 45 | 60 | 70 | 123 | 71 | 25 | 34 | 30 | 93 | 97 | 57 | 15 | 70 | 59 | 78 |
|  | **nA** | 11 | 13 | 13 | 13 | 12 | 13 | 8 | 10 | 12 | 12 | 11 | 11 | 13 | 11 | 12 | 11 | 12 | 11 | 12 | 8 | 12 | 10 | 10 | 7 | 9 | 9 | 12 |
|  | **HO** | 0.80 | 0.85 | 0.87 | 0.74 | 0.78 | 0.79 | 0.69 | 0.79 | 0.66 | 0.80 | 0.67 | 0.74 | 0.78 | 0.65 | 0.7 | 0.76 | 0.87 | 0.8 | 0.76 | 0.77 | 0.77 | 0.74 | 0.70 | 0.67 | 0.67 | 0.76 | 0.71 |
|  | **HE** | 0.81 | 0.84 | 0.85 | 0.81 | 0.80 | 0.83* | 0.75 | 0.86 | 0.83* | 0.85 | 0.77 | 0.86 | 0.85 | 0.86* | 0.83 | 0.82 | 0.86 | 0.84 | 0.88 | 0.84 | 0.85 | 0.82 | 0.80 | 0.80 | 0.80 | 0.78 | 0.81 |
|  | **FIS** | 0.01 | -0.02 | -0.02 | 0.08 | 0.02 | 0.05 | 0 | 0.14 | 0.21 | 0.07 | 0.14 | 0.14 | 0.08 | 0.25* | 0.16 | 0.07 | -0.01 | 0.04 | 0.13 | 0.09 | 0.09 | 0.1 | 0.13 | 0.17 | 0.17 | 0.02 | 0.13 |
| **C12** | **n** | 41 | 80 | 90 | 88 | 47 | 85 | 29 | 19 | 56 | 44 | 21 | 50 | 45 | 60 | 70 | 123 | 71 | 25 | 34 | 30 | 96 | 97 | 57 | 15 | 71 | 59 | 79 |
|  | **nA** | 7 | 7 | 7 | 8 | 8 | 7 | 6 | 5 | 6 | 8 | 7 | 7 | 7 | 8 | 7 | 9 | 8 | 5 | 7 | 4 | 8 | 7 | 6 | 5 | 5 | 4 | 6 |
|  | **HO** | 0.61 | 0.7 | 0.71 | 0.65 | 0.64 | 0.72 | 0.52 | 0.63 | 0.83 | 0.75 | 0.62 | 0.80 | 0.67 | 0.65 | 0.76 | 0.61 | 0.72 | 0.60 | 0.76 | 0.57 | 0.66 | 0.70 | 0.74 | 0.67 | 0.63 | 0.27 | 0.49 |
|  | **HE** | 0.67 | 0.62 | 0.68 | 0.64 | 0.63 | 0.71 | 0.68 | 0.68 | 0.67 | 0.70 | 0.65 | 0.72 | 0.67 | 0.67 | 0.68 | 0.68 | 0.67 | 0.70 | 0.74 | 0.56 | 0.64 | 0.66 | 0.71 | 0.71 | 0.59 | 0.30 | 0.54 |
|  | **FIS** | 0.09 | -0.13 | -0.04 | -0.01 | -0.02 | -0.01 | 0.12 | 0.1 | -0.03 | -0.08 | 0.05 | -0.12 | 0 | 0.03 | -0.12 | 0.1 | -0.08 | 0.15 | -0.03 | -0.01 | -0.03 | -0.06 | -0.04 | 0.06 | -0.08 | 0.1 | 0.08 |
| **I9** | **n** | 41 | 80 | 90 | 88 | 46 | 86 | 29 | 19 | 56 | 44 | 21 | 50 | 44 | 59 | 69 | 123 | 70 | 25 | 34 | 30 | 80 | 97 | 57 | 15 | 70 | 59 | 79 |
|  | **nA** | 7 | 10 | 10 | 10 | 8 | 9 | 6 | 5 | 10 | 8 | 6 | 9 | 7 | 8 | 9 | 11 | 8 | 7 | 8 | 6 | 8 | 10 | 9 | 6 | 5 | 6 | 5 |
|  | **HO** | 0.83 | 0.83 | 0.82 | 0.77 | 0.74 | 0.78 | 0.90 | 0.89 | 0.86 | 0.86 | 0.86 | 0.80 | 0.75 | 0.71 | 0.70 | 0.75 | 0.81 | 0.84 | 0.85 | 0.87 | 0.83 | 0.79 | 0.77 | 1 | 0.79 | 0.86 | 0.68 |
|  | **HE** | 0.80 | 0.81 | 0.78 | 0.80 | 0.77 | 0.81 | 0.79 | 0.73 | 0.80 | 0.82 | 0.74 | 0.79 | 0.79 | 0.80 | 0.78 | 0.79 | 0.79 | 0.81 | 0.81 | 0.77 | 0.80 | 0.80 | 0.78 | 0.83 | 0.72 | 0.75 | 0.71 |
|  | **FIS** | -0.04 | -0.02 | -0.05 | 0.04 | 0.05 | 0.04 | -0.2 | -0.22 | -0.07 | -0.05 | -0.16 | -0.02 | 0.06 | 0.11 | 0.11 | 0.05 | -0.04 | -0.04 | -0.06 | -0.13 | -0.04 | 0 | 0.01 | -0.21 | -0.10 | -0.16 | 0.04 |
| **E4** | **n** | 40 | 79 | 90 | 89 | 47 | 86 | 29 | 19 | 56 | 44 | 21 | 50 | 46 | 60 | 70 | 124 | 71 | 25 | 34 | 30 | 94 | 97 | 57 | 15 | 71 | 59 | 79 |
|  | **nA** | 10 | 12 | 12 | 13 | 12 | 12 | 10 | 10 | 11 | 11 | 9 | 11 | 10 | 11 | 10 | 14 | 13 | 9 | 12 | 9 | 13 | 11 | 10 | 8 | 9 | 10 | 9 |
|  | **HO** | 0.90 | 0.85 | 0.84 | 0.84 | 0.85 | 0.92 | 0.93 | 0.84 | 0.86 | 0.89 | 0.76 | 0.86 | 0.7 | 0.90 | 0.81 | 0.87 | 0.90 | 0.92 | 0.85 | 0.77 | 0.85 | 0.89 | 0.79 | 0.93 | 0.72 | 0.85 | 0.91 |
|  | **HE** | 0.87 | 0.88 | 0.86 | 0.87 | 0.86 | 0.88 | 0.87 | 0.89 | 0.87 | 0.89 | 0.86 | 0.88 | 0.86* | 0.89 | 0.88 | 0.88 | 0.87 | 0.87 | 0.86 | 0.78 | 0.89 | 0.85 | 0.81 | 0.83 | 0.85 | 0.76 | 0.85 |
|  | **FIS** | -0.03 | 0.04 | 0.02 | 0.03 | 0.01 | -0.05 | -0.07 | 0.12 | 0.02 | -0.01 | 0.12 | 0.02 | 0.20 | -0.02 | 0.07 | 0.01 | -0.03 | -0.07 | 0.01 | 0.02 | 0.04 | -0.04 | 0.02 | -0.13 | 0.03 | -0.11 | -0.07 |
| **A5** | **n** | 41 | 79 | 90 | 89 | 47 | 86 | 29 | 19 | 56 | 42 | 21 | 50 | 45 | 60 | 70 | 124 | 71 | 25 | 34 | 30 | 96 | 97 | 57 | 15 | 71 | 59 | 79 |
|  | **nA** | 4 | 5 | 4 | 4 | 5 | 5 | 3 | 3 | 4 | 4 | 4 | 6 | 4 | 5 | 5 | 5 | 4 | 5 | 5 | 6 | 6 | 5 | 4 | 4 | 6 | 5 | 6 |
|  | **HO** | 0.71 | 0.62 | 0.69 | 0.67 | 0.60 | 0.71 | 0.52 | 0.32 | 0.68 | 0.62 | 0.67 | 0.42 | 0.56 | 0.53 | 0.73 | 0.52 | 0.63 | 0.56 | 0.71 | 0.7 | 0.58 | 0.64 | 0.74 | 0.8 | 0.75 | 0.56 | 0.75 |
|  | **HE** | 0.65 | 0.65 | 0.60 | 0.62 | 0.53 | 0.67 | 0.57 | 0.49 | 0.71 | 0.74 | 0.60 | 0.69* | 0.72 | 0.69* | 0.61 | 0.69 | 0.72 | 0.74 | 0.74 | 0.72 | 0.60* | 0.72 | 0.65 | 0.67 | 0.77 | 0.70 | 0.70 |
|  | **FIS** | -0.09 | 0.05 | -0.14 | -0.09 | -0.13 | -0.05 | 0.05 | 0.36 | 0.04 | 0.16 | -0.11 | 0.39* | 0.23 | 0.23 | -0.19 | 0.24* | 0.12 | 0.24 | 0.05 | 0.03 | 0.03 | 0.12 | -0.13 | -0.2 | 0.04 | 0.2 | -0.06 |
| **M5** | **n** | 41 | 79 | 90 | 89 | 46 | 85 | 29 | 19 | 56 | 44 | 21 | 50 | 46 | 60 | 68 | 124 | 71 | 25 | 33 | 30 | 96 | 97 | 57 | 15 | 70 | 59 | 77 |
|  | **nA** | 19 | 24 | 22 | 25 | 20 | 19 | 19 | 16 | 22 | 16 | 13 | 21 | 20 | 24 | 22 | 27 | 24 | 16 | 16 | 11 | 17 | 18 | 14 | 9 | 12 | 10 | 13 |
|  | **HO** | 0.95 | 0.86 | 0.86 | 0.87 | 0.85 | 0.85 | 0.74 | 0.84 | 0.89 | 0.89 | 0.90 | 0.82 | 0.87 | 0.85 | 0.72 | 0.83 | 0.79 | 0.96 | 0.73 | 0.72 | 0.85 | 0.86 | 0.75 | 1 | 0.80 | 0.86 | 0.84 |
|  | **HE** | 0.90 | 0.86 | 0.86 | 0.87 | 0.87 | 0.84 | 0.87* | 0.86 | 0.84 | 0.88 | 0.79 | 0.85 | 0.87 | 0.86 | 0.84 | 0.88 | 0.87 | 0.88 | 0.81 | 0.86 | 0.87 | 0.84 | 0.78 | 0.85 | 0.83 | 0.69 | 0.84 |
|  | **FIS** | -0.06 | 0.01 | 0 | 0 | 0.03 | -0.01 | 0.16 | -0.04 | -0.06 | -0.01 | -0.15 | 0.04 | 0 | 0.01 | 0.14 | 0.06 | 0.09 | -0.09 | 0.10 | 0.11 | 0.02 | -0.02 | 0.03 | -0.18 | 0.04 | -0.25 | -0.01 |
| **L11** | **n** | 41 | 80 | 90 | 89 | 47 | 86 | 29 | 19 | 56 | 44 | 21 | 50 | 46 | 60 | 70 | 124 | 71 | 25 | 34 | 30 | 96 | 97 | 57 | 30 | 71 | 59 | 78 |
|  | **nA** | 4 | 7 | 6 | 7 | 5 | 6 | 6 | 5 | 5 | 6 | 5 | 7 | 5 | 5 | 5 | 7 | 8 | 4 | 6 | 5 | 5 | 6 | 5 | 4 | 5 | 4 | 4 |
|  | **HO** | 0.76 | 0.64 | 0.67 | 0.69 | 0.70 | 0.76 | 0.72 | 0.53 | 0.71 | 0.64 | 0.57 | 0.64 | 0.65 | 0.68 | 0.84 | 0.67 | 0.69 | 0.56 | 0.71 | 0.73 | 0.77 | 0.64 | 0.86 | 0.80 | 0.72 | 0.68 | 0.64 |
|  | **HE** | 0.72 | 0.70 | 0.7 | 0.69 | 0.68 | 0.72 | 0.70 | 0.71 | 0.68 | 0.72 | 0.70 | 0.74 | 0.70 | 0.67 | 0.70 | 0.70 | 0.71 | 0.71 | 0.73 | 0.72 | 0.70 | 0.65 | 0.77 | 0.70 | 0.69 | 0.73 | 0.69 |
|  | **FIS** | -0.05 | 0.09 | 0.05 | 0 | -0.04 | -0.06 | 0.03 | 0.2 | -0.06 | 0.12 | 0.19 | 0.13 | 0.07 | -0.02 | -0.2 | 0.04 | 0.02 | 0.22 | 0.03 | -0.02 | -0.11 | 0.02 | -0.12 | -0.15 | -0.04 | 0.07 | 0.07 |
| **J1** | **n** | 41 | 80 | 90 | 89 | 47 | 86 | 29 | 19 | 56 | 44 | 21 | 50 | 46 | 59 | 70 | 124 | 71 | 25 | 34 | 30 | 96 | 97 | 57 | 15 | 71 | 58 | 79 |
|  | **nA** | 10 | 17 | 15 | 17 | 15 | 15 | 14 | 12 | 15 | 15 | 9 | 15 | 13 | 14 | 15 | 17 | 13 | 10 | 12 | 7 | 14 | 15 | 14 | 9 | 11 | 9 | 11 |
|  | **HO** | 0.80 | 0.81 | 0.80 | 0.87 | 0.89 | 0.81 | 0.83 | 0.89 | 0.86 | 0.82 | 0.81 | 0.82 | 0.85 | 0.95 | 0.80 | 0.84 | 0.77 | 0.76 | 0.76 | 0.83 | 0.91 | 0.75 | 0.86 | 0.8 | 0.7 | 0.86 | 0.77 |
|  | **HE** | 0.85 | 0.89 | 0.89 | 0.90 | 0.91 | 0.89 | 0.89 | 0.90 | 0.84 | 0.85 | 0.84 | 0.85 | 0.85 | 0.89 | 0.89 | 0.84 | 0.84 | 0.87 | 0.85 | 0.83 | 0.90 | 0.86* | 0.87 | 0.85 | 0.81 | 0.82 | 0.83 |
|  | **FIS** | 0.06 | 0.09 | 0.10 | 0.04 | 0.01 | 0.08 | 0.02 | -0.01 | -0.02 | 0.04 | 0.04 | 0.03 | 0 | -0.06 | 0.1 | 0.01 | 0.08 | 0.12 | 0.10 | 0 | -0.01 | 0.12 | 0.01 | 0.06 | 0.14 | -0.06 | 0.07 |
| **G2** | **n** | 41 | 79 | 89 | 89 | 46 | 85 | 29 | 19 | 56 | 43 | 21 | 50 | 46 | 59 | 67 | 124 | 71 | 25 | 34 | 27 | 94 | 97 | 56 | 15 | 69 | 59 | 79 |
|  | **nA** | 16 | 16 | 22 | 19 | 17 | 20 | 13 | 13 | 17 | 15 | 15 | 17 | 18 | 16 | 18 | 21 | 19 | 12 | 17 | 11 | 19 | 15 | 15 | 10 | 14 | 14 | 14 |
|  | **HO** | 0.93 | 0.87 | 0.90 | 0.88 | 0.98 | 0.89 | 0.79 | 0.79 | 0.84 | 0.84 | 0.86 | 0.76 | 0.87 | 0.85 | 0.87 | 0.83 | 0.87 | 0.88 | 0.82 | 0.63 | 0.90 | 0.70 | 0.73 | 0.73 | 0.64 | 0.71 | 0.78 |
|  | **HE** | 0.92 | 0.89 | 0.92 | 0.89 | 0.90 | 0.90 | 0.90* | 0.91 | 0.89 | 0.88 | 0.92 | 0.88 | 0.91 | 0.89 | 0.88 | 0.90* | 0.90 | 0.90 | 0.90 | 0.82 | 0.87 | 0.74 | 0.80 | 0.67 | 0.88* | 0.79 | 0.83 |
|  | **FIS** | -0.01 | 0.02 | 0.02 | 0.02 | -0.09 | 0 | 0.08 | 0.19 | 0.06 | 0.05 | 0.07 | 0.14 | 0.04 | 0.05 | 0.02 | 0.08 | 0.04 | 0.02 | 0.09 | 0.24 | -0.04 | 0.06 | 0.09 | -0.1 | 0.28* | 0.1 | 0.05 |
| **G3** | **n** | 41 | 79 | 90 | 89 | 46 | 86 | 29 | 19 | 55 | 44 | 21 | 50 | 45 | 59 | 70 | 124 | 70 | 23 | 34 | 30 | 92 | 97 | 57 | 15 | 71 | 59 | 79 |
|  | **nA** | 7 | 7 | 9 | 8 | 9 | 6 | 6 | 5 | 7 | 7 | 4 | 5 | 7 | 7 | 8 | 8 | 10 | 5 | 5 | 3 | 4 | 6 | 6 | 4 | 4 | 4 | 7 |
|  | **HO** | 0.46 | 0.53 | 0.42 | 0.39 | 0.48 | 0.38 | 0.48 | 0.68 | 0.42 | 0.43 | 0.24 | 0.42 | 0.47 | 0.24 | 0.57 | 0.46 | 0.49 | 0.39 | 0.32 | 0.4 | 0.45 | 0.38 | 0.28 | 0.47 | 0.25 | 0.37 | 0.44 |
|  | **HE** | 0.68* | 0.66* | 0.61* | 0.59* | 0.68* | 0.63* | 0.65 | 0.71 | 0.66* | 0.67* | 0.67* | 0.65* | 0.63 | 0.57* | 0.69 | 0.68* | 0.67 | 0.59 | 0.56* | 0.37 | 0.57 | 0.51* | 0.41 | 0.61 | 0.54* | 0.52 | 0.58 |
|  | **FIS** | 0.32 | 0.20 | 0.31 | 0.33* | 0.3 | 0.39* | 0.35 | 0.04 | 0.37 | 0.36 | 0.65* | 0.36 | 0.26 | 0.58* | 0.18 | 0.33* | 0.28 | 0.35 | 0.43 | -0.08 | 0.22 | 0.26 | 0.32 | 0.24 | 0.53* | 0.28 | 0.24 |
| **I8** | **n** | 41 | 78 | 90 | 89 | 47 | 86 | 29 | 19 | 56 | 44 | 21 | 50 | 46 | 59 | 70 | 123 | 71 | 25 | 34 | 30 | 93 | 97 | 55 | 15 | 71 | 59 | 79 |
|  | **nA** | 14 | 12 | 12 | 12 | 10 | 13 | 10 | 10 | 13 | 11 | 9 | 11 | 10 | 11 | 11 | 13 | 11 | 11 | 13 | 7 | 14 | 10 | 10 | 6 | 9 | 9 | 9 |
|  | **HO** | 0.76 | 0.82 | 0.80 | 0.80 | 0.74 | 0.86 | 0.86 | 0.84 | 0.77 | 0.77 | 0.76 | 0.88 | 0.67 | 0.75 | 0.84 | 0.80 | 0.86 | 0.92 | 0.79 | 0.8 | 0.84 | 0.81 | 0.65 | 0.93 | 0.82 | 0.71 | 0.85 |
|  | **HE** | 0.82 | 0.81 | 0.79 | 0.82 | 0.79 | 0.82 | 0.82 | 0.81 | 0.83 | 0.79 | 0.85 | 0.85 | 0.74 | 0.80 | 0.85 | 0.80 | 0.81* | 0.85 | 0.79 | 0.83 | 0.83 | 0.83 | 0.74 | 0.83 | 0.83 | 0.86 | 0.81 |
|  | **FIS** | 0.08 | -0.01 | -0.02 | 0.03 | 0.05 | -0.05 | -0.03 | -0.05 | 0.07 | 0.03 | 0.11 | -0.04 | 0.09 | 0.07 | 0 | 0.01 | -0.06 | -0.08 | -0.01 | 0.04 | -0.01 | 0.01 | 0.11 | -0.13 | 0.02 | 0.17 | -0.05 |
| **L7** | **n** | 41 | 80 | 90 | 89 | 47 | 86 | 29 | 19 | 56 | 44 | 21 | 50 | 46 | 60 | 70 | 124 | 71 | 25 | 34 | 30 | 96 | 97 | 57 | 15 | 71 | 59 | 79 |
|  | **nA** | 4 | 4 | 4 | 4 | 5 | 4 | 3 | 3 | 4 | 4 | 3 | 4 | 4 | 4 | 5 | 5 | 4 | 3 | 4 | 3 | 4 | 3 | 3 | 3 | 4 | 3 | 4 |
|  | **HO** | 0.44 | 0.53 | 0.54 | 0.48 | 0.55 | 0.42 | 0.34 | 0.53 | 0.55 | 0.64 | 0.43 | 0.42 | 0.57 | 0.38 | 0.54 | 0.52 | 0.41 | 0.60 | 0.53 | 0.6 | 0.46 | 0.48 | 0.51 | 0.67 | 0.54 | 0.61 | 0.51 |
|  | **HE** | 0.58 | 0.61 | 0.6 | 0.62 | 0.61 | 0.63* | 0.65* | 0.62 | 0.6* | 0.62 | 0.56 | 0.57 | 0.61 | 0.62* | 0.68 | 0.60* | 0.65* | 0.51 | 0.59 | 0.58 | 0.59* | 0.66* | 0.65 | 0.63 | 0.62* | 0.58 | 0.56 |
|  | **FIS** | 0.25 | 0.15 | 0.1 | 0.23 | 0.1 | 0.34* | 0.51 | 0.13 | 0.08 | -0.02 | 0.23 | 0.27 | 0.08 | 0.38 | 0.21 | 0.13 | 0.37* | -0.17 | 0.1 | -0.04 | 0.22 | 0.27 | 0.22 | -0.07 | 0.13 | -0.06 | 0.09 |
| **H5** | **n** | 41 | 80 | 90 | 88 | 46 | 85 | 29 | 19 | 56 | 44 | 21 | 50 | 46 | 59 | 70 | 124 | 71 | 25 | 33 | 30 | 91 | 97 | 57 | 15 | 71 | 59 | 78 |
|  | **nA** | 14 | 16 | 17 | 17 | 16 | 18 | 14 | 17 | 19 | 15 | 13 | 18 | 17 | 16 | 17 | 19 | 17 | 13 | 16 | 11 | 18 | 15 | 16 | 11 | 11 | 12 | 13 |
|  | **HO** | 0.85 | 0.89 | 0.88 | 0.90 | 0.78 | 0.81 | 0.93 | 0.95 | 0.86 | 0.86 | 0.90 | 0.88 | 0.80 | 0.85 | 0.83 | 0.80 | 0.83 | 0.88 | 0.85 | 0.63 | 0.95 | 0.92 | 0.95 | 0.93 | 0.73 | 0.85 | 0.86 |
|  | **HE** | 0.88 | 0.87 | 0.85 | 0.88 | 0.86 | 0.87 | 0.89 | 0.90 | 0.90 | 0.89 | 0.88 | 0.90 | 0.87 | 0.86 | 0.87 | 0.89 | 0.9 | 0.88 | 0.89 | 0.79 | 0.88 | 0.84 | 0.85 | 0.85 | 0.86* | 0.89 | 0.83 |
|  | **FIS** | 0.03 | -0.02 | -0.03 | -0.02 | 0.09 | 0.08 | 0.09 | -0.04 | 0.05 | 0.03 | -0.03 | 0.02 | 0.07 | 0.02 | 0.05 | 0.11 | 0.07 | 0 | 0.05 | 0.20 | -0.08 | -0.1 | -0.11 | -0.1 | 0.15 | 0.04 | -0.04 |
| **B6** | **n** | 41 | 80 | 90 | 87 | 46 | 84 | 29 | 19 | 54 | 43 | 21 | 50 | 44 | 59 | 68 | 124 | 71 | 24 | 33 | 30 | 80 | 97 | 57 | 15 | 70 | 57 | 78 |
|  | **nA** | 15 | 19 | 21 | 19 | 15 | 18 | 14 | 15 | 16 | 16 | 13 | 15 | 15 | 19 | 20 | 19 | 16 | 15 | 16 | 10 | 18 | 15 | 14 | 11 | 11 | 12 | 12 |
|  | **HO** | 0.93 | 0.96 | 0.94 | 0.91 | 0.91 | 0.94 | 0.9 | 1 | 0.96 | 0.98 | 1 | 0.94 | 0.80 | 0.95 | 0.90 | 0.9 | 0.93 | 0.83 | 0.94 | 0.70 | 0.96 | 0.91 | 0.84 | 0.93 | 0.83 | 0.86 | 0.82 |
|  | **HE** | 0.92 | 0.92 | 0.92 | 0.92 | 0.91 | 0.93 | 0.92 | 0.94 | 0.92 | 0.92 | 0.91 | 0.92 | 0.92 | 0.92 | 0.92 | 0.92 | 0.92 | 0.92 | 0.93 | 0.77 | 0.91 | 0.85 | 0.88 | 0.89 | 0.85 | 0.85 | 0.80 |
|  | **FIS** | -0.01 | -0.04 | -0.02 | 0.02 | -0.01 | -0.01 | 0.01 | -0.07 | -0.04 | -0.06 | -0.11 | -0.02 | 0.14 | -0.03 | 0.02 | 0.03 | -0.02 | 0.1 | -0.01 | 0.10 | -0.05 | -0.07 | 0.05 | -0.05 | 0.03 | -0.01 | -0.03 |
| **F4** | **n** | 41 | 80 | 90 | 89 | 47 | 85 | 29 | 19 | 56 | 44 | 21 | 50 | 46 | 58 | 70 | 123 | 71 | 25 | 34 | 30 | 96 | 97 | 56 | 15 | 71 | 59 | 79 |
|  | **nA** | 8 | 10 | 11 | 12 | 9 | 10 | 10 | 7 | 9 | 8 | 8 | 11 | 9 | 11 | 11 | 10 | 6 | 7 | 8 | 5 | 10 | 9 | 9 | 6 | 9 | 10 | 8 |
|  | **HO** | 0.76 | 0.71 | 0.78 | 0.78 | 0.81 | 0.73 | 0.66 | 0.58 | 0.80 | 0.80 | 0.76 | 0.72 | 0.78 | 0.79 | 0.77 | 0.80 | 0.80 | 0.92 | 0.82 | 0.73 | 0.68 | 0.72 | 0.79 | 1 | 0.77 | 0.63 | 0.78 |
|  | **HE** | 0.79 | 0.76 | 0.81 | 0.81 | 0.79 | 0.80 | 0.81 | 0.79 | 0.79 | 0.79 | 0.80 | 0.80 | 0.79 | 0.77 | 0.83 | 0.79 | 0.81 | 0.80 | 0.84 | 0.72 | 0.80 | 0.77 | 0.8 | 0.76 | 0.8 | 0.64 | 0.78 |
|  | **FIS** | 0.04 | 0.06 | 0.04 | 0.05 | -0.02 | 0.09 | 0.26 | 0.25 | -0.02 | -0.01 | 0.05 | 0.10 | 0.01 | -0.03 | 0.07 | -0.02 | 0 | -0.15 | 0.02 | -0.01 | 0.16 | 0.06 | 0.02 | -0.33 | 0.04 | 0.02 | -0.01 |
| **C10** | **n** | 41 | 80 | 90 | 89 | 47 | 84 | 29 | 19 | 56 | 44 | 21 | 50 | 45 | 60 | 68 | 124 | 71 | 25 | 34 | 30 | 93 | 97 | 57 | 15 | 70 | 59 | 77 |
|  | **nA** | 21 | 20 | 20 | 21 | 16 | 21 | 14 | 14 | 21 | 19 | 16 | 19 | 18 | 21 | 17 | 21 | 20 | 16 | 18 | 9 | 20 | 17 | 17 | 12 | 10 | 9 | 12 |
|  | **HO** | 0.90 | 0.84 | 0.72 | 0.81 | 0.74 | 0.88 | 0.72 | 0.95 | 0.82 | 0.7 | 0.86 | 0.86 | 0.91 | 0.88 | 0.78 | 0.85 | 0.79 | 0.84 | 0.82 | 0.77 | 0.84 | 0.91 | 0.58 | 0.93 | 0.64 | 0.71 | 0.70 |
|  | **HE** | 0.90 | 0.91 | 0.88 | 0.87 | 0.89 | 0.90 | 0.89* | 0.92 | 0.89 | 0.85 | 0.89 | 0.92 | 0.91 | 0.91 | 0.86 | 0.89 | 0.89 | 0.90 | 0.92 | 0.78 | 0.91 | 0.87 | 0.74* | 0.91 | 0.76 | 0.78 | 0.76 |
|  | **FIS** | -0.01 | 0.09 | 0.18* | 0.07 | 0.16 | 0.02 | 0.17 | -0.04 | 0.08 | 0.18 | 0.04 | 0.07 | -0.01 | 0.03 | 0.09 | 0.05 | 0.11 | 0.06 | 0.10 | 0.02 | 0.08 | -0.04 | 0.22 | -0.03 | 0.16 | 0.09 | 0.08 |

**Supplementary Table S3.** Pearson correlation coefficient in allele frequencies of neutral microsatellites loci between pairs of temporal groups composed of wild populations sampled in 2009, 2015 and 2016.

| **Comparison of wild groups** | **r (x, y)** | **t- test (p-value)** |
| --- | --- | --- |
|  |  |  |
| wild2009 vs wild2015 | 0.551 | 13.27 (*p* < 0.01) |
| wild2009 vs wild2016 | 0.557 | 13.48 (*p* < 0.01) |
| Wild2015 vs wild2016 | 0.988 | 129.2 (*p* < 0.001) |

**Supplementary Table S4.** Statistical power of used markers for detecting various true levels of population differentiation (*F*_ST_) by means of Fisher's exact test simulated in the program POWSIM when using allele frequencies of the dataset presented in this study.

| ***F*_ST_** | **Effective** | **Generations** | **19 microsatellite loci** | **Mean of individual microsatellite loci** |
| --- | --- | --- | --- | --- |
|  | **size (N*e*)** | **of drift (t)** |  |  |
|  |  |  | **Fisher’s exact test** | **Fisher’s exact test** |
| 0.0012 | 2000 | 5 | 1 |  |
| 0.0025 | 1000 | 5 | 1 | 0.845 |
| 0.005 | 1000 | 10 | 1 | 0.986 |
| 0.01 | 1000 | 20 | 1 |  |
| 0.025 | 2000 | 10 | 1 |  |

**Supplementary Table S5.** Pairwise *F*_ST_ values based on 19 neutral microsatellites loci among 27 populations of gilthead seabream from the Adriatic Sea, including wild, farmed, farmed associated and wild YOY populations. Significant *F*_ST_ values are underlined at p < 0.0001 (Bonferroni correction). Population codes are explained in Tables 1. Coloured cells presents significant pair-wise comparisons, where light pink indicates lower *F*_ST_ values (0.006-0.01), pink medium *F*_ST_ values (0.01 – 0.05) and red higher *F*_ST_ values (above 0.05).

|  | **09**  **WH** | **15**  **WT** | **15**  **WN** | **16**  **WR** | **16**  **WU** | **16**  **WV** | **16**  **WK** | **16**  **WD** | **15**  **AK** | **15**  **AB** | **15**  **AV** | **16**  **AK** | **16**  **AB** | **17**  **AK** | **17**  **AB** | **15**  **JP** | **15**  **JN** | **16**  **JP** | **16**  **JR** | **16**  **JB** | **15**  **FC** | **15**  **FI** | **16**  **FI** | **16**  **FG** | **15**  **FF** | **16**  **FFa** |
| --- | --- | --- | --- | --- | --- | --- | --- | --- | --- | --- | --- | --- | --- | --- | --- | --- | --- | --- | --- | --- | --- | --- | --- | --- | --- | --- |
| **15WT** | 0.071 |  |  |  |  |  |  |  |  |  |  |  |  |  |  |  |  |  |  |  |  |  |  |  |  |  |
| **15WN** | 0.076 | 0.001 |  |  |  |  |  |  |  |  |  |  |  |  |  |  |  |  |  |  |  |  |  |  |  |  |
| **16WR** | 0.076 | 0.001 | 0.001 |  |  |  |  |  |  |  |  |  |  |  |  |  |  |  |  |  |  |  |  |  |  |  |
| **16WU** | 0.071 | 0.002 | 0.001 | 0.003 |  |  |  |  |  |  |  |  |  |  |  |  |  |  |  |  |  |  |  |  |  |  |
| **16WV** | 0.071 | 0.001 | 0.001 | 0.000 | 0.002 |  |  |  |  |  |  |  |  |  |  |  |  |  |  |  |  |  | Legend | | |  |
| **16WK** | 0.077 | 0.002 | 0.004 | 0.001 | 0.004 | 0.005 |  |  |  |  |  |  |  |  |  |  |  |  |  |  |  |  |  | 0.006 |  |  |
| **16WD** | 0.064 | 0.000 | 0.001 | 0.002 | -0.002 | 0.002 | 0.002 |  |  |  |  |  |  |  |  |  |  |  |  |  |  |  |  | 0.010 |  |  |
| **15AK** | 0.075 | 0.006 | 0.008 | 0.009 | 0.011 | 0.006 | 0.012 | 0.009 |  |  |  |  |  |  |  |  |  |  |  |  |  |  |  | 0.050 |  |  |
| **15AB** | 0.075 | 0.008 | 0.011 | 0.012 | 0.012 | 0.009 | 0.013 | 0.014 | 0.001 |  |  |  |  |  |  |  |  |  |  |  |  |  |  | 0.100 |  |  |
| **15AV** | 0.076 | 0.011 | 0.012 | 0.014 | 0.006 | 0.012 | 0.017 | 0.003 | 0.002 | 0.005 |  |  |  |  |  |  |  |  |  |  |  |  |  |  |  |  |
| **16AK** | 0.069 | 0.007 | 0.006 | 0.008 | 0.008 | 0.007 | 0.009 | 0.006 | 0.001 | 0.003 | 0.002 |  |  |  |  |  |  |  |  |  |  |  |  |  |  |  |
| **16AB** | 0.076 | 0.007 | 0.007 | 0.009 | 0.012 | 0.006 | 0.014 | 0.012 | 0.000 | 0.000 | 0.006 | 0.002 |  |  |  |  |  |  |  |  |  |  |  |  |  |  |
| **17AK** | 0.082 | 0.007 | 0.009 | 0.008 | 0.014 | 0.010 | 0.011 | 0.007 | 0.020 | 0.024 | 0.030 | 0.020 | 0.019 |  |  |  |  |  |  |  |  |  |  |  |  |  |
| **17AB** | 0.072 | 0.003 | 0.001 | 0.003 | 0.005 | 0.003 | 0.006 | 0.001 | 0.011 | 0.017 | 0.019 | 0.011 | 0.013 | 0.004 |  |  |  |  |  |  |  |  |  |  |  |  |
| **15JP** | 0.072 | 0.008 | 0.008 | 0.010 | 0.008 | 0.008 | 0.012 | 0.007 | 0.001 | 0.001 | 0.000 | 0.001 | 0.001 | 0.023 | 0.014 |  |  |  |  |  |  |  |  |  |  |  |
| **15JN** | 0.073 | 0.009 | 0.011 | 0.010 | 0.011 | 0.009 | 0.016 | 0.012 | 0.002 | 0.000 | 0.004 | 0.001 | 0.001 | 0.022 | 0.015 | 0.001 |  |  |  |  |  |  |  |  |  |  |
| **16JP** | 0.073 | 0.007 | 0.007 | 0.007 | 0.012 | 0.006 | 0.010 | 0.010 | 0.001 | 0.002 | 0.006 | -0.002 | -0.001 | 0.019 | 0.014 | -0.001 | 0.003 |  |  |  |  |  |  |  |  |  |
| **16JR** | 0.078 | 0.012 | 0.009 | 0.016 | 0.015 | 0.009 | 0.019 | 0.015 | 0.002 | 0.003 | 0.009 | 0.003 | 0.000 | 0.023 | 0.016 | 0.006 | 0.004 | 0.003 |  |  |  |  |  |  |  |  |
| **16JB** | 0.102 | 0.032 | 0.032 | 0.034 | 0.036 | 0.030 | 0.042 | 0.034 | 0.027 | 0.029 | 0.032 | 0.032 | 0.030 | 0.041 | 0.034 | 0.029 | 0.028 | 0.033 | 0.030 |  |  |  |  |  |  |  |
| **15FC** | 0.083 | 0.011 | 0.014 | 0.011 | 0.014 | 0.014 | 0.014 | 0.010 | 0.018 | 0.021 | 0.022 | 0.019 | 0.019 | 0.006 | 0.010 | 0.019 | 0.023 | 0.019 | 0.027 | 0.043 |  |  |  |  |  |  |
| **15FI** | 0.092 | 0.026 | 0.028 | 0.024 | 0.027 | 0.023 | 0.035 | 0.027 | 0.027 | 0.028 | 0.034 | 0.035 | 0.025 | 0.024 | 0.024 | 0.031 | 0.029 | 0.034 | 0.036 | 0.048 | 0.027 |  |  |  |  |  |
| **16FI** | 0.107 | 0.028 | 0.024 | 0.024 | 0.028 | 0.023 | 0.031 | 0.028 | 0.033 | 0.033 | 0.037 | 0.036 | 0.034 | 0.024 | 0.024 | 0.034 | 0.038 | 0.035 | 0.039 | 0.053 | 0.028 | 0.033 |  |  |  |  |
| **16FG** | 0.094 | 0.024 | 0.027 | 0.025 | 0.031 | 0.021 | 0.031 | 0.025 | 0.033 | 0.038 | 0.049 | 0.040 | 0.033 | 0.015 | 0.017 | 0.039 | 0.040 | 0.037 | 0.042 | 0.063 | 0.021 | 0.009 | 0.030 |  |  |  |
| **15FF** | 0.091 | 0.027 | 0.029 | 0.030 | 0.033 | 0.025 | 0.035 | 0.028 | 0.021 | 0.021 | 0.023 | 0.023 | 0.022 | 0.039 | 0.030 | 0.023 | 0.021 | 0.025 | 0.021 | 0.006 | 0.041 | 0.044 | 0.056 | 0.058 |  |  |
| **16FFa** | 0.108 | 0.040 | 0.045 | 0.046 | 0.046 | 0.045 | 0.048 | 0.047 | 0.035 | 0.037 | 0.035 | 0.035 | 0.038 | 0.054 | 0.047 | 0.038 | 0.037 | 0.041 | 0.041 | 0.032 | 0.051 | 0.067 | 0.074 | 0.076 | 0.026 |  |
| **16FFb** | 0.092 | 0.024 | 0.025 | 0.026 | 0.027 | 0.023 | 0.029 | 0.027 | 0.035 | 0.038 | 0.041 | 0.036 | 0.037 | 0.033 | 0.021 | 0.037 | 0.039 | 0.040 | 0.041 | 0.025 | 0.039 | 0.044 | 0.053 | 0.053 | 0.020 | 0.045 |

**Supplementary Table S6.** Overall performance of model-based Bayesian methods, implemented in the software packages NewHybrids and Structure, to correctly identify individuals simulated using the R package hybriddetective^89^ as admixed under different proportions of hybrids in the datasets (15%, 33%, 66%). Yellow highlights the thresholds that perform well. Due to the reduced ability of microsatellites to detect backcross hybrid classes at high probabilities, BF1 and BF2 were not presented separately and were instead merged within the category Hybrid all.

| a) Performance - 15 % hybrids | | | |  |  | |  |  |  |  |  |  |
| --- | --- | --- | --- | --- | --- | --- | --- | --- | --- | --- | --- | --- |
|  |  |  | Probability threshold | | | | | | | | | |
|  |  | NewHybrids | |  | Structure | | | | | | | |
|  |  | *0.50* | |  | *0.60* | | *0.70* | *0.75* | *0.80* | *0.85* | *0.90* | *0.95* |
| Pure Wild |  | 0.96 | |  | 0.90 | | 0.93 | 0.90 | 0.96 | 0.97 | 0.98 | 0.98 |
| Pure Farmed |  | 0.96 | |  | 0.89 | | 0.93 | 0.89 | 0.94 | 0.96 | 0.96 | 0.94 |
| Hybrids F1_F2 |  | 0.66 | |  | 0.41 | | 0.66 | 0.77 | 0.83 | 0.91 | 0.90 | 0.55 |
| Average |  | **0.86** | |  | **0.74** | | **0.84** | **0.85** | **0.91** | **0.95** | **0.94** | **0.82** |
|  |  |  | |  |  | |  |  |  |  |  |  |
|  |  | 0.50 | |  | 0.60 | | 0.70 | 0.75 | 0.80 | 0.85 | 0.90 | 0.95 |
| Pure Wild |  | 0.96 | |  | 0.90 | | 0.93 | 0.90 | 0.96 | 0.97 | 0.98 | 0.98 |
| Pure Farmed |  | 0.96 | |  | 0.89 | | 0.93 | 0.89 | 0.94 | 0.96 | 0.96 | 0.94 |
| Hybrid all |  | 0.72 | |  | 0.26 | | 0.48 | 0.59 | 0.66 | 0.76 | 0.80 | 0.76 |
| Average |  | **0.88** | |  | **0.69** | | **0.78** | **0.79** | **0.85** | **0.91** | **0.91** | **0.89** |
|  |  |  | |  |  | |  |  |  |  |  |  |
| b) Performance - 33% hybrids | | | |  |  | |  |  |  |  |  |  |
|  |  |  | Probability threshold | | | | | | | | | |
|  |  | NewHybrids | |  |  | Structure | | | | | | |
|  |  | *0.50* | |  | *0.60* | | *0.70* | *0.75* | *0.80* | *0.85* | *0.90* | *0.95* |
| Pure Wild |  | 0.86 | |  | 0.74 | | 0.79 | 0.83 | 0.84 | 0.86 | 0.84 | 0.74 |
| Pure Farmed |  | 0.86 | |  | 0.75 | | 0.81 | 0.84 | 0.88 | 0.92 | 0.92 | 0.88 |
| Hybrids F1_F2 |  | 0.83 | |  | 0.52 | | 0.74 | 0.84 | 0.91 | 0.89 | 0.83 | 0.59 |
| Average |  | **0.85** | |  | **0.67** | | **0.78** | **0.84** | **0.88** | **0.89** | **0.86** | **0.73** |
|  |  |  | |  |  | |  |  |  |  |  |  |
|  |  | 0.50 | |  | 0.60 | | 0.70 | 0.75 | 0.80 | 0.85 | 0.90 | 0.95 |
| Pure Wild |  | 0.86 | |  | 0.74 | | 0.79 | 0.83 | 0.84 | 0.86 | 0.84 | 0.74 |
| Pure Farmed |  | 0.86 | |  | 0.75 | | 0.81 | 0.84 | 0.88 | 0.92 | 0.92 | 0.88 |
| Hybrid all |  | 0.69 | |  | 0.32 | | 0.50 | 0.61 | 0.69 | 0.76 | 0.76 | 0.70 |
| Average |  | **0.80** | |  | **0.60** | | **0.70** | **0.76** | **0.80** | **0.84** | **0.84** | **0.77** |
|  |  |  | |  |  | |  |  |  |  |  |  |
| c) Performance - 66% hybrids | | | |  |  | |  |  |  |  |  |  |
|  |  |  | Probability threshold | | | | | | | | | |
|  |  | NewHybrids | |  |  | Structure | | | | | | |
|  |  | 0.50 | |  | 0.60 | | 0.70 | 0.75 | 0.80 | 0.85 | 0.90 | 0.95 |
| Pure Wild |  | 0.58 | |  | 0.42 | | 0.52 | 0.56 | 0.65 | 0.72 | 0.70 | 0.18 |
| Pure Farmed |  | 0.66 | |  | 0.40 | | 0.51 | 0.56 | 0.60 | 0.67 | 0.65 | 0.15 |
| Hybrids F1_F2 |  | 0.84 | |  | 0.43 | | 0.76 | 0.86 | 0.92 | 0.92 | 0.85 | 0.55 |
| Average |  | **0.69** | |  | **0.42** | | **0.60** | **0.66** | **0.72** | **0.77** | **0.73** | **0.29** |
|  |  |  | |  |  | |  |  |  |  |  |  |
|  |  | 0.50 | |  | 0.60 | | 0.70 | 0.75 | 0.80 | 0.85 | 0.90 | 0.95 |
| Pure Wild |  | 0.58 | |  | 0.42 | | 0.52 | 0.56 | 0.65 | 0.72 | 0.70 | 0.18 |
| Pure Farmed |  | 0.66 | |  | 0.40 | | 0.51 | 0.56 | 0.60 | 0.67 | 0.65 | 0.15 |
| Hybrid all |  | 0.71 | |  | 0.29 | | 0.53 | 0.62 | 0.71 | 0.79 | 0.83 | 0.70 |
| Average |  | **0.65** | |  | **0.37** | | **0.52** | **0.58** | **0.65** | **0.73** | **0.73** | **0.35** |

| (i) | | 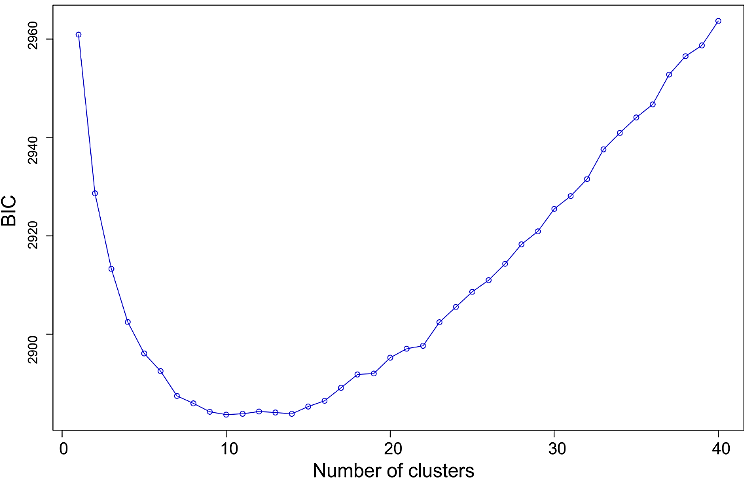 |
| --- | --- | --- |
|  | | |
| (ii) | 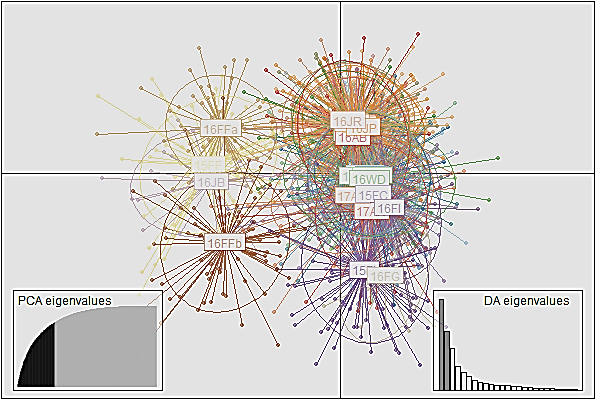 | |
| **Supplementary** **Figure S1**. The upper figure (i) shows the selection of the optimal number of clusters in the lowest Bayesian Information Criterion (BIC), while the lower figure (ii) shows the scatterplot of the final Discriminant Analysis of Principal Components (DAPC) model with sampling locality as a prior, where points are individual genotypes, colour-coded by their original sampling locality and surrounded by a 95% confidence ellipse. DA and PCA Eigenvalues represent the amount of genetic variation captured by the analysis, and the first two discriminant factors are plotted as the x- and y- axis. | | |

| 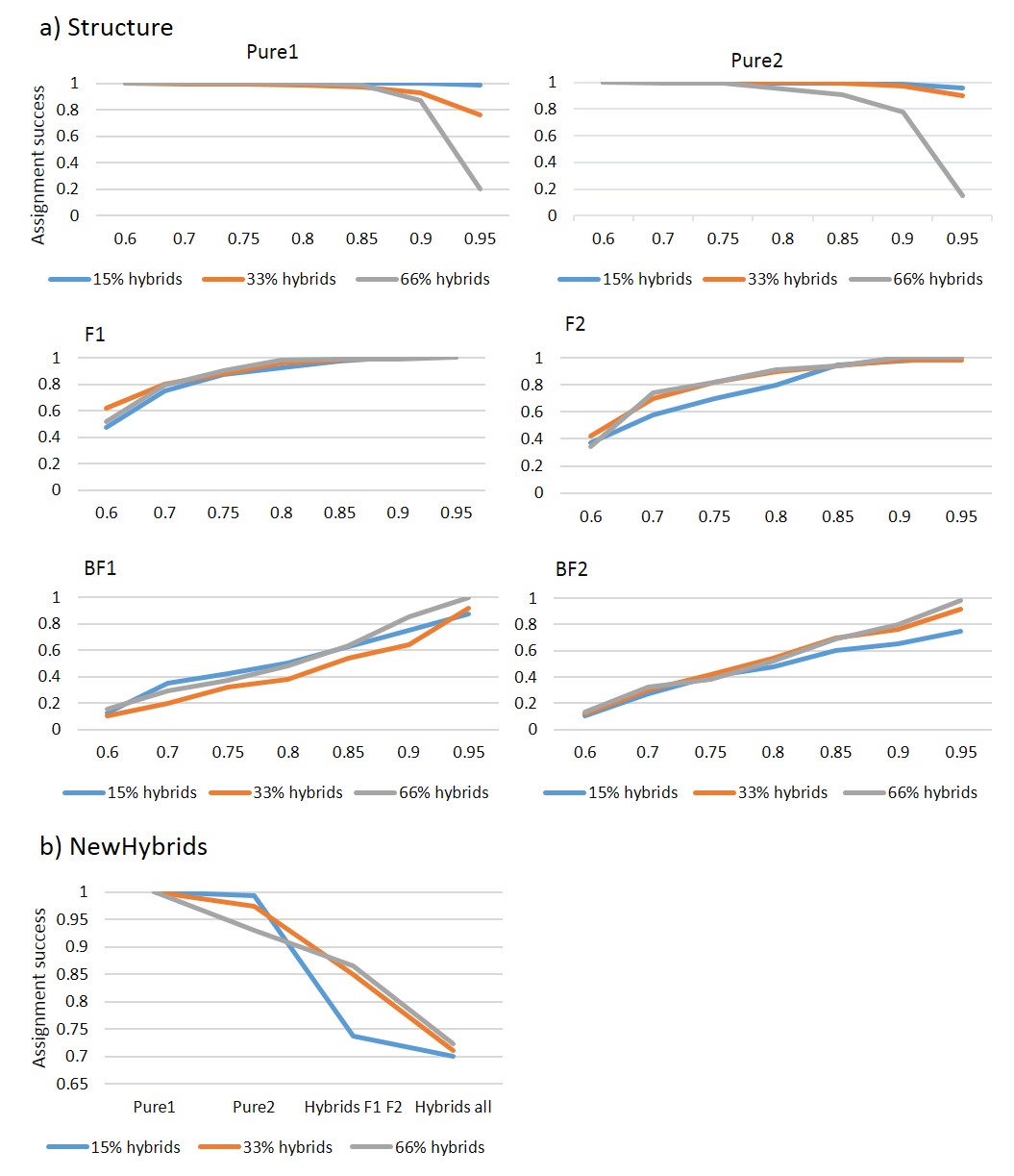 |
| --- |
| **Supplementary** **Figure S2.** Hybrid detection efficiency of (a) Structure and (b) NewHybrids for each genotype class with variable hybrids proportions in the samples (15%, 33%, 66%) based on simulated individual genotypes of 19 microsatellite loci used. For NewHybrids, only the posterior probability value of 50% was used as a threshold for assigning individuals to that specific class. Posterior probability values for all hybrid classes for an individual were summed and used as one estimate for the category *Hybrids all*. |

**Supplementary Methods:**

**ROMS model setups**

Two different ROMS setups were used to calculate input fields for IBM (hourly averaged current, temperature and salinity fields) for the period from 1 January to 15 May 2016. The first ROMS setup (Adriatic ROMS) is identical to that used in Džoić et al.^54^, with the domain covering the entire Adriatic Sea with a resolution of 2.5 km. In the second ROMS setup (ASHELF2 ROMS^32^) a smaller domain was used, encompassing the eastern coastal area of the middle Adriatic, with a resolution of 1 km (Figure 1). The small domain was used to a gain more realistic and detailed reproduction of circulation in a complex topographic area. Horizontal scalar fields with a resolution of 8 km (air pressure, air temperature, relative humidity, cloudiness, precipitation and shortwave radiation) and vector fields with a resolution of 2 km (wind) from the operational ALADIN model^90^ were used to force both ROMS models together with tides, river inflows and water mass exchange across open boundaries. Both ROMS sea surface currents (SSC) were validated with SSC measured by HF radars located at the islands Brač and Vis (Figure 1) (<http://www.izor.hr/nascum/index_eng.htm>).

**Modelled ROMS circulation and HF radar verification**

Looking at the sea surface current fields, we can see that the Adriatic ROMS model successfully reproduced the main currents in the Adriatic Sea (Supplementary Figure S3). The Western Adriatic Current (WAC) outflows from the Adriatic Sea are confined to the Italian coast and the Eastern Adriatic Current (EAC) flows in the NW direction along the Croatian coast^91^. During February (Supplementary Figure S3a) and March (Supplementary Figure S3b), EAC was strengthened due to the dominant sirocco winds blowing from the SE^92,93^. The Southern Adriatic Gyre was also successfully reproduced^91^.

| 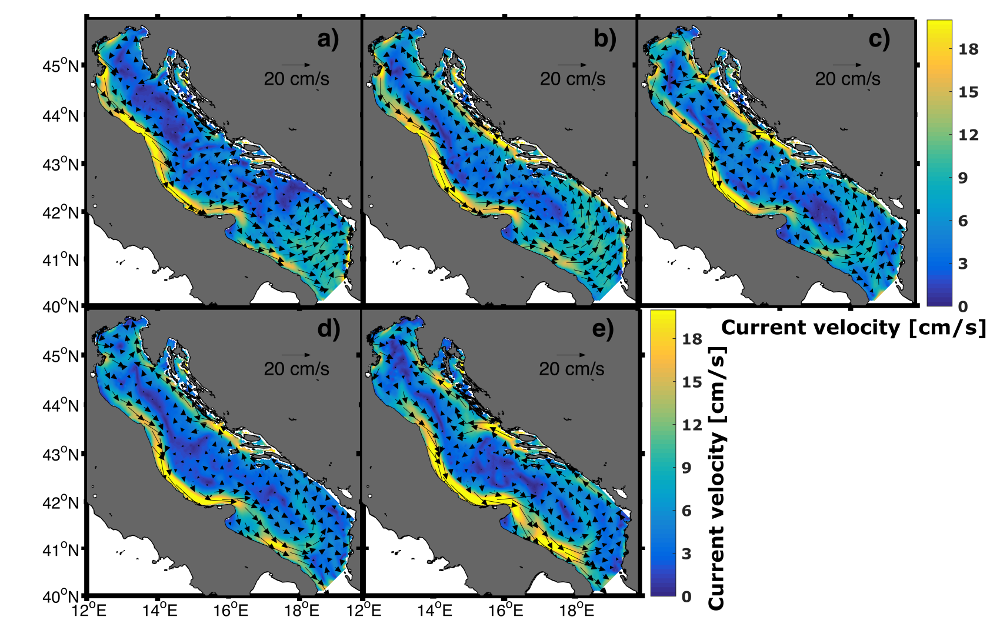 |
| --- |
| **Supplementary** **Figure S3.** Monthly mean sea surface currents calculated by Adriatic ROMS for January (a), February (b), March (c), April (d) and the first 15 days of May (e) 2016. Vectors are plotted at every tenth grid point. The figure has been created using MATLAB 2014a ([www.mathworks.com](http://www.mathworks.com/)) and GIMP 2.8.16 ([www.gimp.org](http://www.gimp.org/)) software. |

Varying atmospheric forcing superimposed on quasi-stationary thermohaline circulation influences the surface current fields calculated by ROMS ASHELF2. Although inflow direction (NW) mostly prevailed throughout the simulated period (Supplementary Figure S4), during February (Supplementary Figure S4c) it was more prominent because of the strong sirocco winds. The monthly averaged sea surface currents measured by HF radars showed a prevailing N-NW direction (Supplementary Figure 5a, 6a, 7a, 8a and 9a). Currents calculated by ASHELF2 ROMS (Supplementary Figure S5b, S6b, S7b, S8b and S9b) more closely matched the HF radars than the Adriatic ROMS (Supplementary Figure S5c, S6c, S7c, S8c and S9c), although general the N-NW inflow direction was obtained in both model setups.

To obtain more detailed insight into the dynamics hidden within monthly averages, the daily averaged current fields were shown for strong bora and sirocco regimes. During a strong bora wind on 22 January 2016 (Supplementary Figure S10a), the ASHELF2 ROMS setup more closely matched the HF radars (Supplementary Figure S10b, S10c) than the Adriatic ROMS setup (Supplementary Figure S10d). During the strong sirocco wind on 8 March 2016 (Supplementary Figure S11a), the N-NW current direction in the western part of the radar domain and small gyre in the upper right corner (Supplementary Figure S11b) were better reproduced by ASHELF2 ROMS (Supplementary Figure S11c) than by the Adriatic ROMS setup (Supplementary Figure S11d).

| 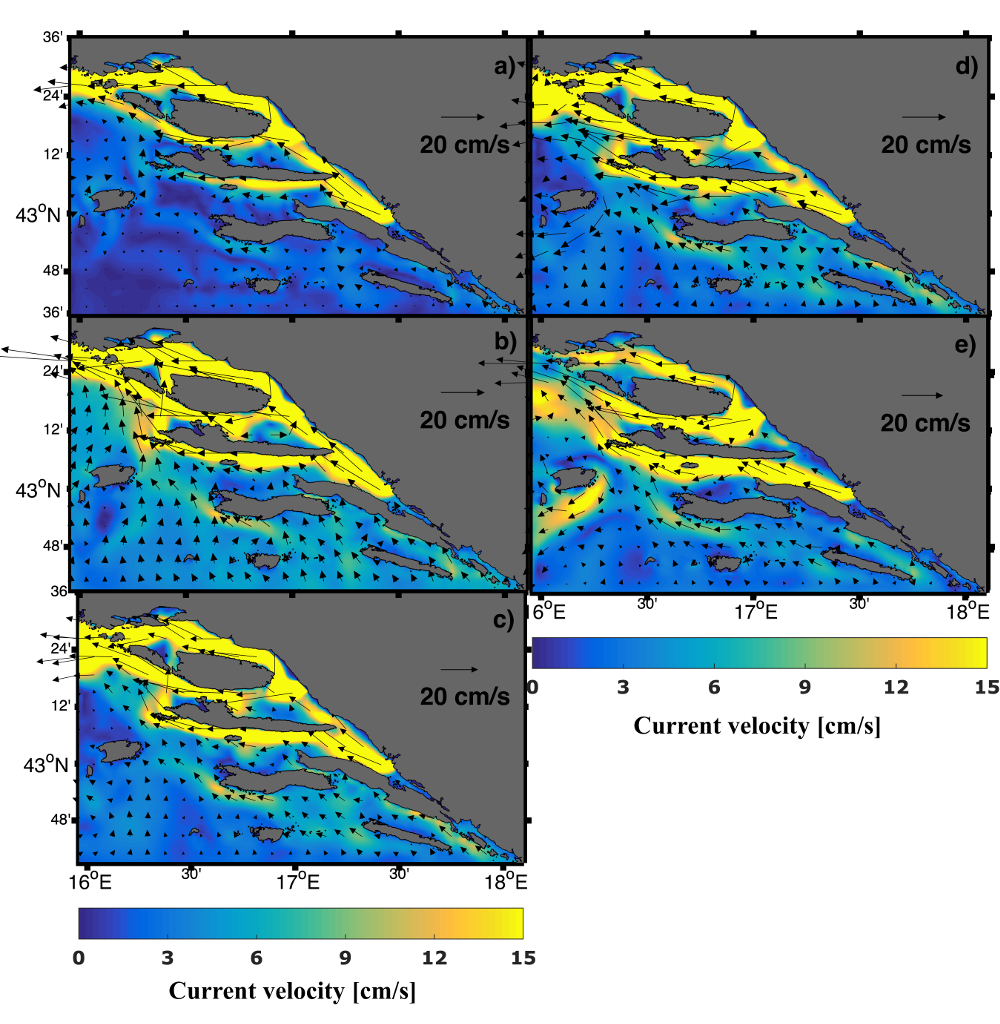 |
| --- |
| **Supplementary Figure S4.** Monthly mean sea surface currents calculated by ASHELF2 ROMS for January (a), February (b), March (c), April (d) and the first 15 days of May (e) 2016. Vectors are plotted at every seventh grid point. The figure has been created using MATLAB 2014a ([www.mathworks.com](http://www.mathworks.com/)) and GIMP 2.8.16 ([www.gimp.org](http://www.gimp.org/)) software. |

| 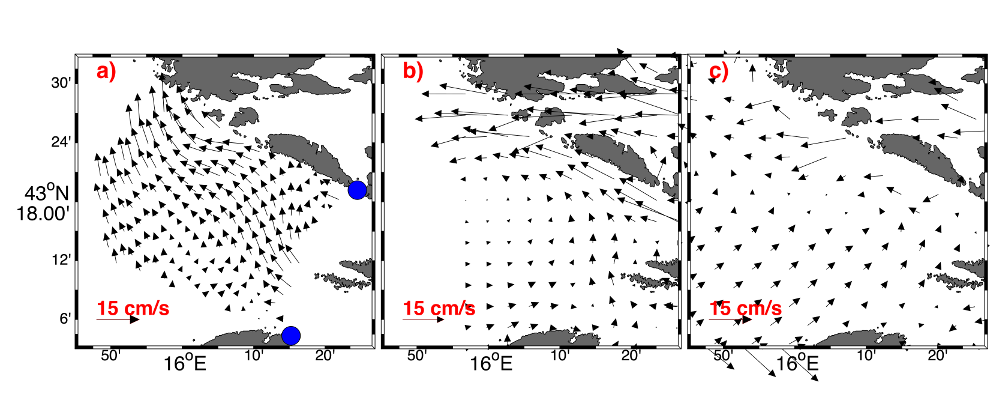 |
| --- |
| **Supplementary Figure S5.** Monthly mean sea surface currents (for January 2016) measured by HF radars (a), calculated by ASHELF2 ROMS (b) and Adriatic ROMS (c). Blue dots are HF radar locations. Radar vectors are plotted at every third grid point, ASHELF2 vectors are plotted at every third grid point and Adriatic vectors are plotted at every second grid point. The figure has been created using MATLAB 2014a ([www.mathworks.com](http://www.mathworks.com/)) and GIMP 2.8.16 ([www.gimp.org](http://www.gimp.org/)) software. |

| 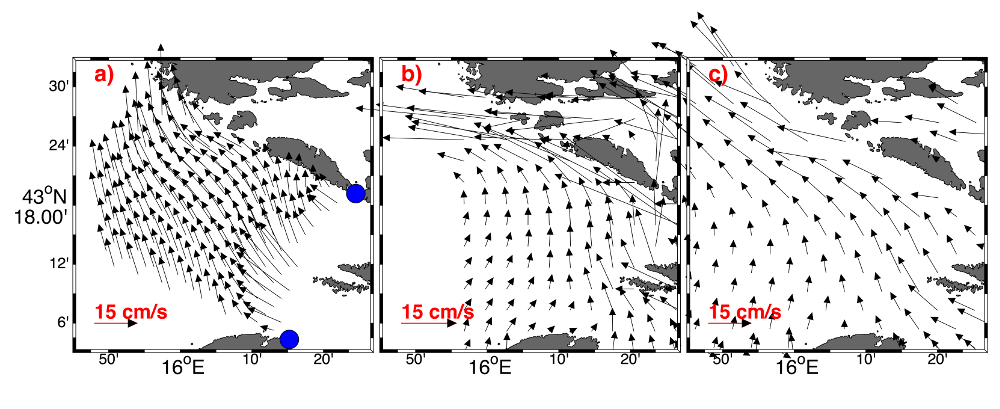 |
| --- |
| **Supplementary Figure S6.** Same as Supplementary Figure S5 but for February 2016. The figure has been created using MATLAB 2014a ([www.mathworks.com](http://www.mathworks.com/)) and GIMP 2.8.16 ([www.gimp.org](http://www.gimp.org/)) software. |

| 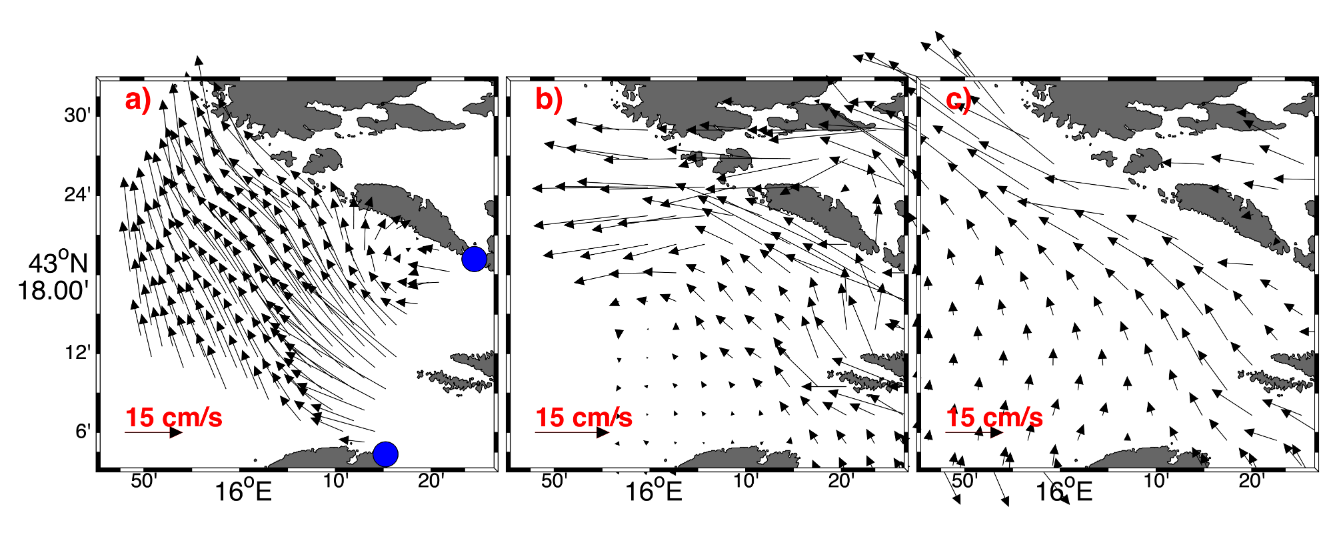 |
| --- |
| **Supplementary Figure S7.** Same as Supplementary Figure S5 but for March 2016. The figure has been created using MATLAB 2014a ([www.mathworks.com](http://www.mathworks.com/)) and GIMP 2.8.16 ([www.gimp.org](http://www.gimp.org/)) software. |

| 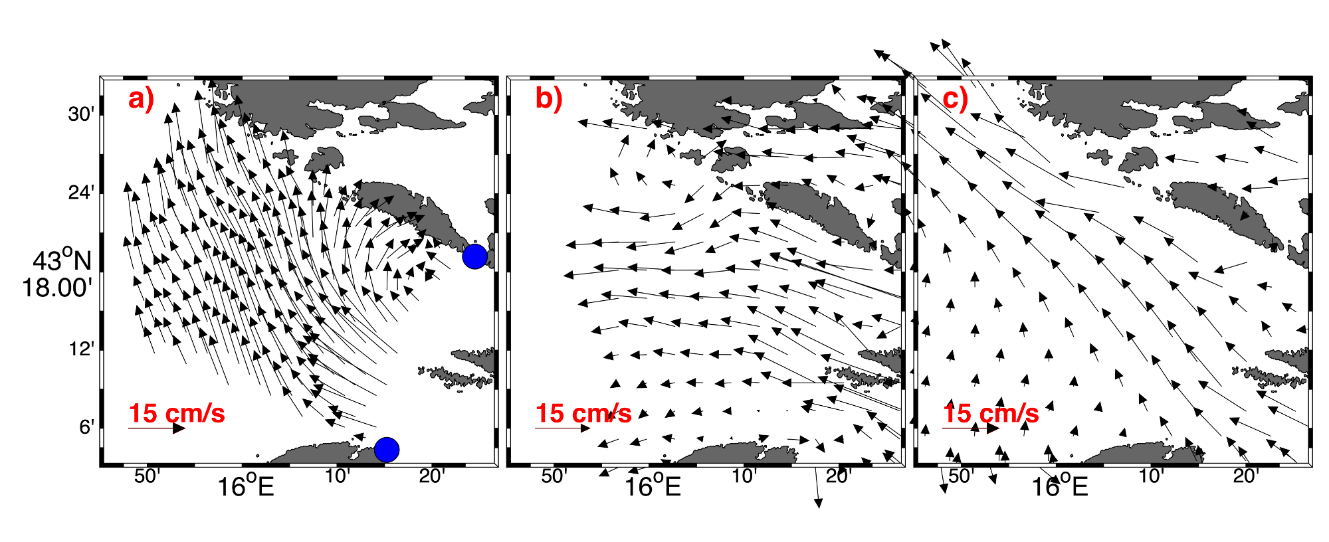 |
| --- |
| **Supplementary Figure S8.** Same as Supplementary Figure S5 but for April 2016. The figure has been created using MATLAB 2014a ([www.mathworks.com](http://www.mathworks.com/)) and GIMP 2.8.16 ([www.gimp.org](http://www.gimp.org/)) software. |

| 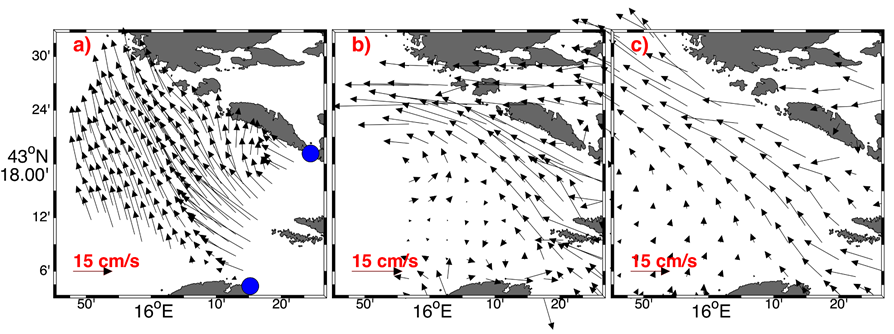 |
| --- |
| **Supplementary Figure S9.** Same as Supplement Figure S3 but for the first half of May 2016. The figure has been created using MATLAB 2014a ([www.mathworks.com](http://www.mathworks.com/)) and GIMP 2.8.16 ([www.gimp.org](http://www.gimp.org/)) software. |

| 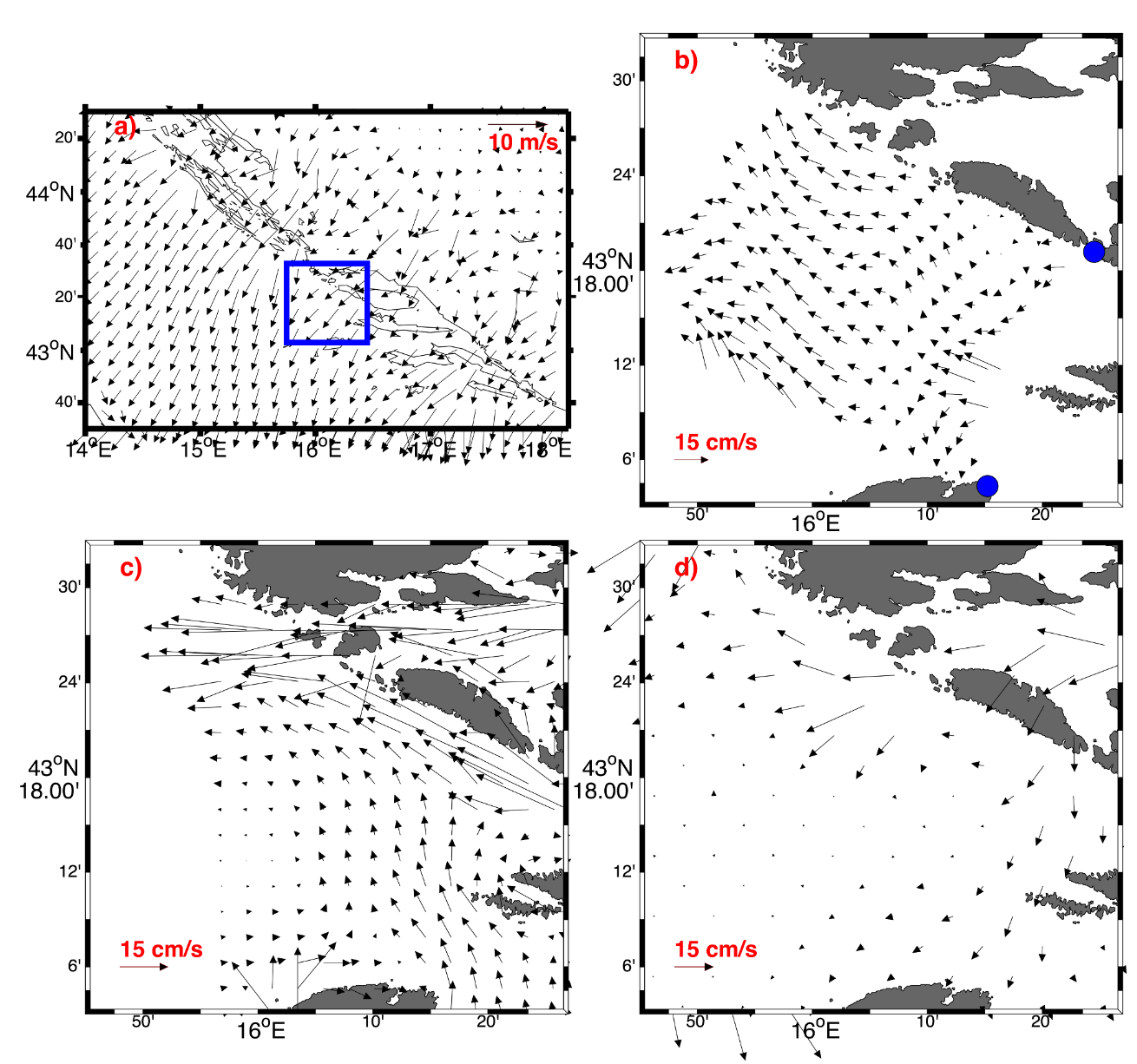 |
| --- |
| **Supplementary Figure S10.** Daily mean wind vectors calculated by ALADIN (a), daily mean sea surface currents measured by HF radars (b), calculated by ASHELF2 ROMS (c) and Adriatic ROMS (d) on 22 January 2016. The blue square in Figure (a) is depicted around the area presented in Figures (b), (c) and (d). Blue dots are HF radar locations (b). Wind vectors are plotted at every seventh grid point, radar vectors are plotted at every third grid point, ASHELF2 vectors are plotted at every third grid point and Adriatic vectors are plotted at every second grid point. The figure has been created using MATLAB 2014a ([www.mathworks.com](http://www.mathworks.com/)) and GIMP 2.8.16 ([www.gimp.org](http://www.gimp.org/)) software. |

| 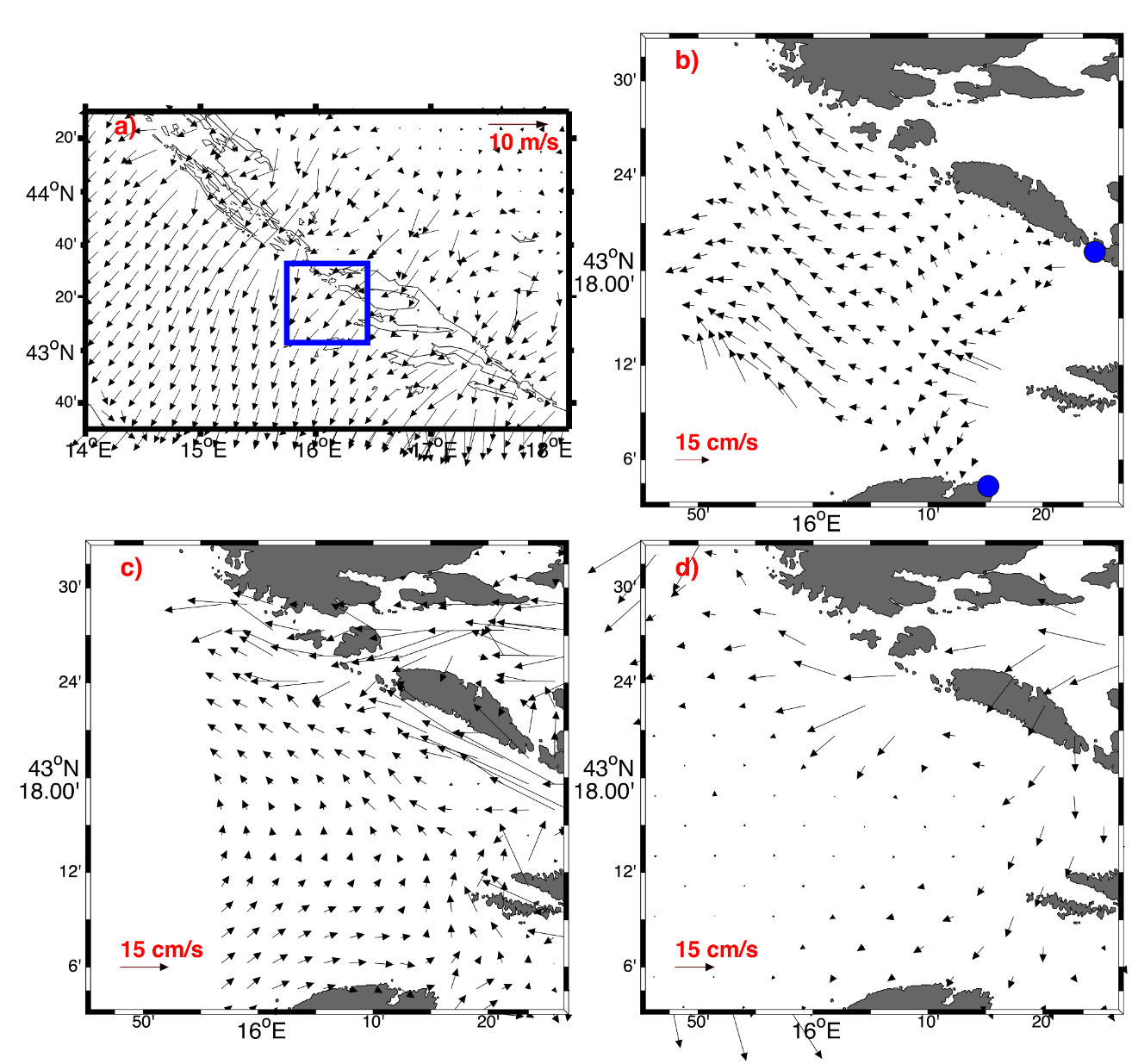 |
| --- |
| **Supplementary Figure S11.** Same as Supplementary Figure S10 but for 8 March 2016. The figure has been created using MATLAB 2014a ([www.mathworks.com](http://www.mathworks.com/)) and GIMP 2.8.16 ([www.gimp.org](http://www.gimp.org/)) software. |

**Supplementary Methods:**

**Ichthyop model setup**

In the IBM experiments, every day at 17 h during the period from 1 January to 29 February, 1000 particles were released from sources at tuna aquaculture areas. Particles were released from stain with a radius of 2 km located at a depth between 30 and 60 m. Spatial particle distribution was calculated at the end of the simulation on 15 May 2016, the date of sampling of the gilthead seabream (16JR – Raša, 16JP – Pantana) in the nursery locations. In the forward dispersion simulations, particles were considered passive drifters. This simplification is acceptable due to the long planktonic stage of gilthead seabream (50 – 70 days^95^), assumed spawning period (1 January to 29 February) and sampling date (15 May). Specimens of gilthead seabream aged 100 days can reach length of 3.5 cm^96^ and they do not swim faster than 0.5-body lengths/s^97^. The average sea surface current speed calculated by Adriatic ROMS for first five months in 2016 was about 10 cm/s. As swimming speed was about 5 times slower than the averaged sea surface current speed, it was neglected.

To test the transport part of gilthead seabream connectivity hypothesis, areas of success were designed as squares of 400 km^2^ with natural nursery ground areas near Pantana and Raša Rivers centrally situated inside (Figure 4). Existence of particles in the area of success at the end of the simulation was the desired outcome. To offset the uneven percentage of sea points in both areas of success, a weighting factor was applied. The number of particles within the square was multiplied by the percentage of sea points within the square. Spatial distributions were calculated by counting the number of particles within the 2.5x2.5 km Adriatic ROMS grid cell for both ROMS setups to obtain uniform visualisation.
